# Natural Variation of a Specific NLR Gene *RGA4L* Confers Strong Chilling Tolerance in Rice

**DOI:** 10.1111/pbi.70293

**Published:** 2025-08-06

**Authors:** Ping Gan, Yongliang Wang, Hanxing Wei, Siyuan Lu, Jinliang Sun, Xianglan Luo, Xiangbing Meng, Peilong Jia, Weijian Cen, Rongbai Li, Hong Yu, Jiayang Li, Jijing Luo

**Affiliations:** ^1^ College of Life Science and Technology, State Key Laboratory for Conservation and Utilization of Subtropical Agro‐Bioresources College of Agriculture, Guangxi University Nanning China; ^2^ State Key Laboratory of Tropical Crop Breeding, Institute of Tropical Bioscience and Biotechnology, and Sanya Research Institute Chinese Academy of Tropical Agricultural Sciences Sanya China; ^3^ Key Laboratory of Seed Innovation Institute of Genetics and Developmental Biology, Chinese Academy of Sciences Beijing China; ^4^ Yazhouwan National Laboratory Sanya China

**Keywords:** chilling tolerance, natural variation, quantitative trait loci, R protein, regulatory mechanism, rice

## Abstract

Low temperature limits rice geographical distribution. However, *japonica* rice, characterised by its chilling tolerance, can be planted in high‐altitude and temperate regions, and the molecular mechanisms underlying this adaptation remain partially understood. Here, we identified a novel major chilling‐tolerant QTL, *qCSS12*, through map‐based cloning using a chromosome segment substitution line (CSSL) previously developed from a cross between the chilling‐tolerant *japonica* rice Koshihikari and the *indica* rice Nona Bokra. Its causal gene *RGA4L* (Disease Resistance Gene Analog 4‐Like) encodes a specific NLR protein. RNAi lines of the Koshihikari allele *RGA4L* ^*jap*^ exhibit increased sensitivity to cold, whereas overexpression lines enhance chilling tolerance of both the vegetative and reproductive stages in rice. Further, we found that RGA4L physically interacts with both OsHSP90 and OsLEA5. These interactions may facilitate the proper assembly of an RGA4L protein complex and then sense and transduce chilling signals to downstream pathways involving *OsLEA5*, thereby conferring chilling tolerance. Moreover, we demonstrate that *RGA4L* has been a major target of artificial selection for low‐temperature acclimation during *japonica* rice domestication. This work shows that *RGA4L* confers chilling tolerance throughout all growth stages and holds potential for breeding cold‐resistant elite rice varieties.

## 1 Introduction

Rice ( *Oryza sativa* L.), which originates from tropical and subtropical regions and is sensitive to chilling stress (Kovach et al. 2007), feeds more than half of the world's population (Zeng et al. 2017). Modern cultivated rice in Asia has evolved into two subspecies—*indica* and *japonica*—which differ in their tolerance to chilling stress (Li et al. 2021). *Japonica* rice is widely cultivated in high‐altitude and temperate regions; therefore, it is more tolerant to chilling than *indica* (Zhang et al. 2017). Despite this, it is a great challenge for rice cultivation to encounter the frequent occurrence of low‐temperature weather. In general, the seedling and reproductive stages of rice (especially booting and flowering) are particularly susceptible to chilling. Statistically, chilling stress is estimated to cause an annual rice yield loss of up to 3–5 million tons in China (Li et al. 2021).

Higher plants, including rice, have evolved sophisticated mechanisms to adapt to adverse environments (Krasensky and Jonak 2012). For instance, the C‐Repeat Binding Factor/Dehydration‐Responsive Element‐Binding Protein 1 (*CBF*/*DREB1*) signalling pathway plays a central role in chilling tolerance in *Arabidopsis* (Chinnusamy et al. 2010; Zhao, Zhang, et al. 2016). The *CBF1* and *CBF3* pathways are strongly induced by chilling stress, and the CBF proteins bind to the promoters of cold‐regulated (COR) genes, thereby enhancing chilling tolerance in *Arabidopsis* (Zhao, Zhang, et al. 2016). In the past decades, only a limited number of quantitative trait loci (QTLs) or genes conferring chilling tolerance have been functionally characterised well in rice (Li et al. 2022; Lu et al. 2019). Among these, *COLD1* contributes to chilling tolerance through interacting with the G‐protein α‐subunit to activate Ca^2+^ channels involved in low temperature sensing (Ma et al. 2015). *CTB4a* can increase the activity of ATP synthase and ATP levels under chilling stress, thus improving chilling tolerance at the booting stage of rice (Zhang et al. 2017). Recently, *IPA1*/*OsSPL14*, a gene previously known for shaping the ideal plant architecture in rice (Miura et al. 2010), was surprisingly also found to be involved in conferring chilling tolerance through the *OsCBF3* signalling pathway as well (Jia et al. 2022).

Plant disease‐resistance (*R*) genes are a group of genes known for their role in plant defence responses (Nguyen et al. 2021; Takken and Goverse 2012). Most plant *R* genes belong to the NB‐LRR (nucleotide‐binding site‐leucine rich repeat) family (NLR), including TNL (Toll/Interleukin‐1 receptor‐nucleotide binding site‐leucine rich repeat) and CNL (coiled‐coil‐nucleotide binding site‐leucine rich repeat) groups, defined by their N‐terminal domains (Lukasik and Takken 2009). The TIR (Toll/interleukin‐1 receptor) and CC (coiled‐coil) domains of NLR proteins are responsible for activating defence signalling (Takken and Goverse 2012; Wang, Hu, et al. 2019), while the central NB‐ARC domain participates in nucleotide binding and ADP to ATP exchange, leading to drastic conformational changes in NLR proteins (Adachi et al. 2019; Wang, Wang, et al. 2019). The C‐terminal LRR domain functions in the direct or indirect recognition of pathogen effectors (Nguyen et al. 2021). In the absence of pathogen effectors, the intra‐molecular interaction of the LRR and NB‐ARC domains maintains the NLR protein in an auto‐inhibition state (off). Upon pathogen attack, effector recognition triggers a conformation change of the NLR proteins, resulting in the disruption of the intra‐molecular interactions and releasing the CC domain to activate downstream defence signalling (on) (Adachi et al. 2019; Takken and Goverse 2012). In recent years, accumulating evidence has suggested that NLR proteins may also play a critical role in the abiotic stress response in higher plants (Ariga et al. 2017; Huang et al. 2010; Liu et al. 2015; Yang et al. 2010). For example, *ACQOS*/*VICTR*, encoding an NLR protein, was suggested to be involved in the trade‐off between abiotic (osmotic tolerance) and biotic stress adaptation (Ariga et al. 2017). Additionally, several other NLR proteins, such as *ADR1*, *CHS2* and *CHS3*, have been shown to significantly influence drought and chilling tolerance in *Arabidopsis*, respectively (Chini et al. 2004; Huang et al. 2010; Yang et al. 2010).

Proper assembly of NLR proteins is known to require the assistance of HSP90 and its co‐chaperones, for example, SGT1 and RAR1 (Adachi et al. 2019; Shirasu 2009; Takken and Goverse 2012; Thao et al. 2007; Wang et al. 2008). Under chilling stress, activation of CHS2 is mediated by the SGT1b‐RAR1‐HSP90 complex and thus triggers the defence response in *Arabidopsis*, leading to chilling sensitivity (Bao et al. 2014). These results highlight the critical role of *HSP90* in response to abiotic/biotic stresses in plants. Meanwhile, a group of late embryogenesis abundant (LEA) family protein‐encoding genes—defined by their pronounced expression during late embryogenesis (Dure et al. 1981)—may be involved in abiotic stress resistance in plants (Huang, Jia, et al. 2018; Huang, Zhang, et al. 2018).

Although great progress has been made in cloning QTLs/genes for chilling tolerance in plants in the past decades, the molecular mechanisms underlying chilling tolerance in rice remain fragmental; meanwhile, none of the previously identified genes were reported to confer chilling tolerance across multiple growth stages. Here, we identified a novel major QTL, designated *qCSS12*, that confers rice chilling tolerance at both the vegetative and reproductive stages. We further demonstrate that a specific NLR gene, *RGA4L*, is the causal gene of *qCSS12*. A 34‐bp mutation in *RGA4L* is responsible for diverging the chilling tolerance of rice. Our study identifies a novel gene for chilling tolerance, with great potential for molecular breeding.

## 2 Results

### 2.1 Identification of a Novel Major QTL *qCSS12* for Chilling Tolerance

**FIGURE 1:**
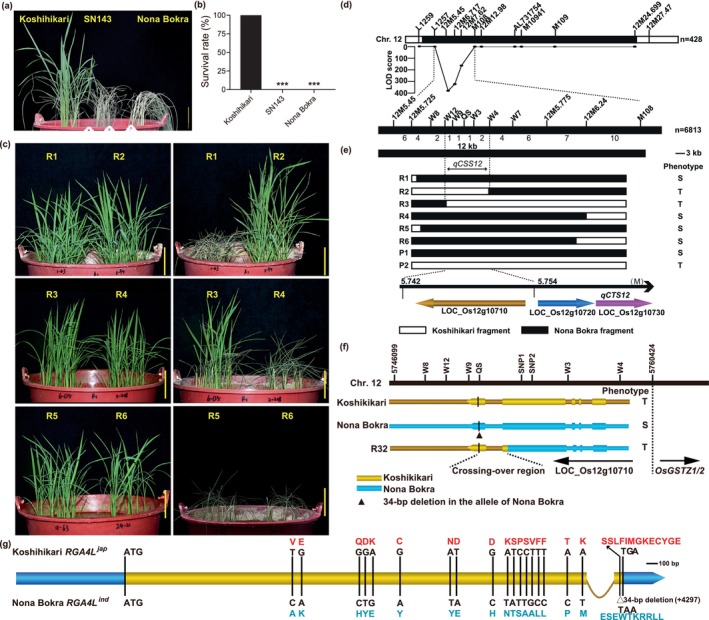
Identification of *qCSS12*. (a, b) Chilling stress phenotypes (a) and survival rate (b) of Koshihikari, SN143, and Nona Bokra. (c) Chilling stress phenotypes of the recombinants. (d) High‐resolution genetic linkage analysis of *qCSS12*. Molecular markers are shown above the chromosome. The number of recombinants between the adjacent markers is indicated under the linkage map. (e) Progeny test of fixed recombinant plants. Chilling tolerance phenotypes of R1 to R6 (corresponding to the ones in c) are shown on the right side. P1 represents Nona Bokra, and P2 represents Koshihikari. The white and black bars represent the homozygous regions of the Koshihikari and Nona Bokra, respectively. (f) Schematic diagram of the crossing‐over region of the recombinant R32. (g) Schematic diagram of gene structure and allelic variations of *RGA4L* between Koshihikari and Nona Bokra. Scale bars, 5 cm. Data are shown as mean ± SD, *n* = 3, ***p < 0.001. R, recombinant; S, chilling‐sensitive; T, chilling‐tolerant.

To isolate genetic loci underlying chilling tolerance of *japonica* rice, we assessed the chilling tolerance phenotypes of a set of chromosome segment substitution lines (CSSLs) previously generated from a cross between the chilling‐tolerant varieties Koshihikari (recurrent parent) and the chilling‐sensitive variety Nona Bokra (donor parent). Among them, SN143, containing a Nona Bokra‐introgressed segment on its chromosome 12, exhibits an obvious chilling‐sensitive phenotype at the seedling stages (Figure 1a,b), suggesting that a major chilling tolerance locus *qCSS12* was identified (Figure S1a). To isolate *qCSS12*, we performed high‐resolution mapping using an F_2_ population of 6813 individuals and successfully narrowed *qCSS12* down to a ~12‐kb region, which contained only a single annotated ORF *LOC_Os12g10710* (Figure 1c–e). Intriguingly, *qCSS12* is closely linked to a previously reported chilling tolerance QTL *qCTS12* (Andaya and Tai 2006), within which *OsGSTZ2* (*LOC_Os12g10730*) is considered to be the causal gene (Kim et al. 2011) (Figure 1e,f). We wondered if *qCSS12* per se is indeed the locus of *qCTS12*. Ultimately, an identified key recombinant R32 ruled out the possibility. A crossover event occurred between the two loci in R32, resulting in a Koshihikari‐derived segment at the 3′‐end and a Nona Bokra segment at the 5′‐end of the crossover region (−strand). The chilling tolerance of R32, therefore, excluded the *qCTS12* locus from the fine‐mapped region of *qCSS12* (Figures 1f and S1b). Sequencing analysis further found that R32 contains the Nona Bokra allele of *OsGSTZ2*, which has a coding sequencing (CDS) identical to the allele derived from chilling‐sensitive variety IR50 (Kim et al. 2011) (Figures 1f and S1c). This indicated that *qCSS12* is a novel major QTL conferring chilling tolerance in rice. However, surprisingly, we could not amplify the cDNA (4647‐bp CDS) of *LOC_Os12g10710* based on the RGAP annotation (http://rice.plantbiology.msu.edu/index.shtml). We, therefore, determined its full‐length cDNA through 5′‐RACE and discovered that the detected cDNA only encompasses the fourth and fifth exons of the RGAP‐annotated *LOC_Os12g10710* with a 4338‐bp coding sequence (Figures 1g and S1d–f). Functional annotation showed that the candidate gene encodes a putative disease‐resistance protein with 100.00% sequence identity to *RGA4* (XP_015619689.1, NCBI). Considering that several genes, such as *LOC_Os01g71114*, *LOC_Os05g31550*, and *LOC_Os11g11790*, have been named *RGA4*, we, therefore, termed the candidate gene *RGA4L*. Sequencing analysis identified 18 non‐synonymous SNPs in the first exon and one indel (34‐bp) in the second exon of the CDSs (Figure 1g). A 34‐bp deletion in the 3′‐end of *RGA4L* ^*ind*^ introduces a premature stop codon, leading to a substantial change in the C‐terminal amino acids (Figure 1g).

### 2.2 *RGA4L* Is a Positive Regulator of Chilling Tolerance in Rice

**FIGURE 2:**
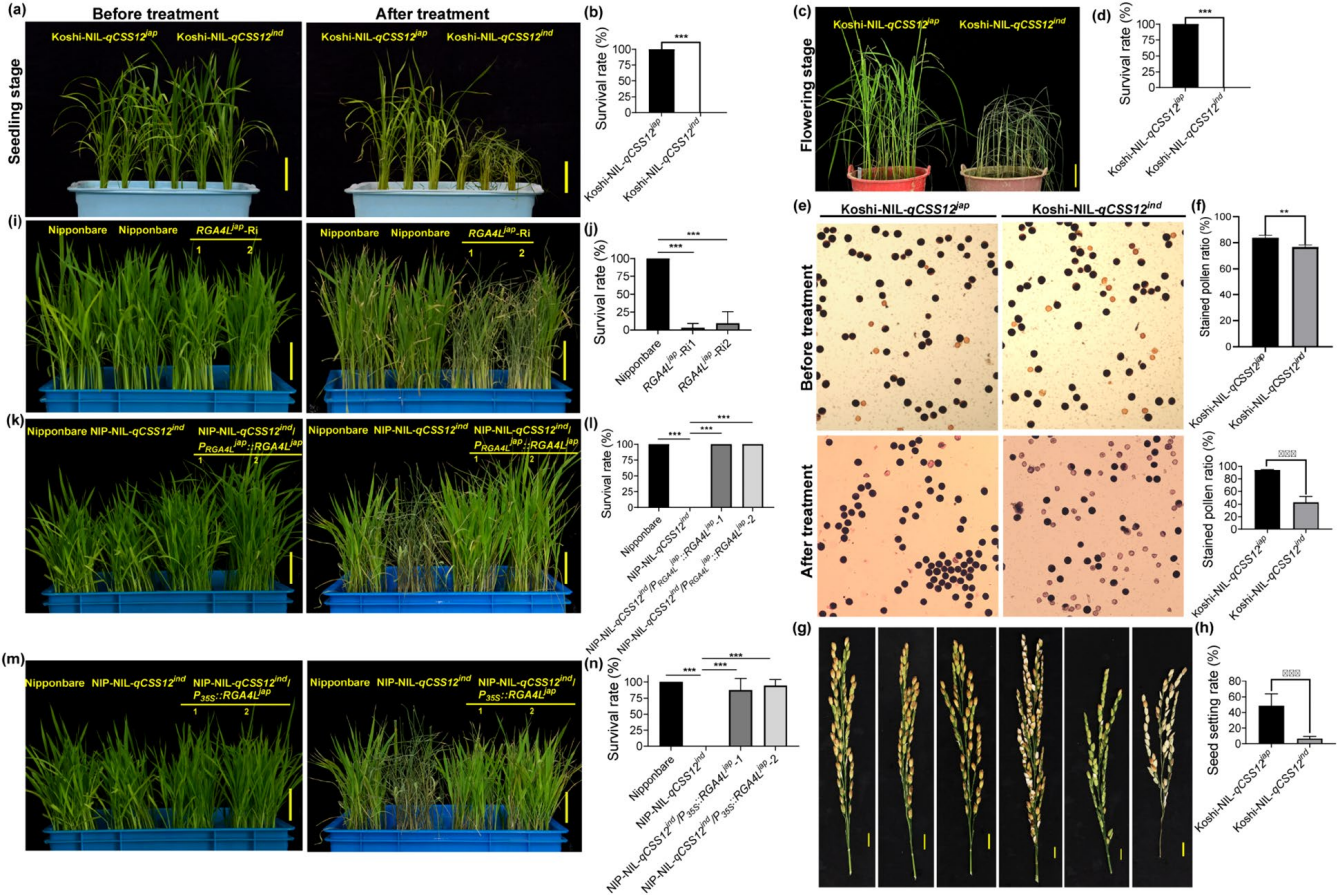
Chilling stress phenotypes of NILs and transgenic lines. (a–f) Phenotypes and survival rates of Koshi‐NIL‐*qCSS12* ^*jap*^ and ‐*qCSS12* ^*ind*^ at the seedling (a, b) and flowering (c, d) stages, respectively. (e, f) Evaluation of pollen fertilities by I_2_–KI staining (e), and comparison of the pollen fertilities of the two genotypes before and after chilling treatment (f). (g, h) Panicles (g) and seed setting rates (h) of Koshi‐NIL‐*qCSS12* ^*jap*^ and ‐*qCSS12* ^*ind*^. (i–n) Phenotypes and survival rates of the *RGA4L* RNAi lines (i, j), the *RGA4L* complementary lines (k, l), and the *RGA4L* overexpressing lines (m, n). Scale bars in (g), 1 cm; scale bars in the rest, 5 cm. Data are shown as mean ± SD, *n* = 5 in (f), *n* = 8 in (h), *n* = 3 for the rest, **p < 0.01, ***p < 0.001.

We then developed nearly isogenic lines (NIL) of *qCSS12* (Koshi‐ and NIP‐NIL‐*qCSS12* ^*ind*^) by introgression of the Nona Bokra locus (*qCSS12* ^*ind*^) into the Koshihikari (Koshi) and Nipponbare (NIP) backgrounds, respectively (Figure S2a). Simultaneously, the *indica* background NILs (NJ11‐, HHZ‐ and 556‐NIL‐*qCSS12* ^*jap*^) were developed by introgression of the Koshihikari locus (*qCSS12* ^*jap*^) into the chilling‐sensitive *indica* varieties, Nanjing 11 (NJ11), Huanghuazhan (HHZ) and 556, respectively (Figure S2b). Correspondingly, compared with their respective controls, Koshi‐ and NIP‐NIL‐*qCSS12* ^*ind*^ exhibited a chilling‐sensitive phenotype similar to that of SN143 (Figures 2a,b,k–n and S2i–k). Intriguingly, *RGA4L* ^*jap*^ also conferred chilling tolerance at the other critical growth stages, including tillering and flowering, during which rice is particularly susceptible to cold injury (Figures 2c–h and S2g,h). Further, we constructed multiple transgenic lines to investigate the function of *RGA4L* (Figure S2c,d). Overall, under normal growth conditions, *RGA4L* ^*jap*^‐native promoter‐driven lines exhibited a slightly rapid growth rate compared to their background line (NIP‐NIL‐*qCSS12* ^*ind*^) and overexpression lines (Figure 2k,m). Phenotypic analysis showed that the *RGA4L* ^*jap*^ knockdown lines exhibited increased chilling sensitivity compared to their wild type Nipponbare (chilling‐tolerant variety) (Figures 2i,j and S2i). In contrast, *RGA4L* ^*jap*^‐native promoter‐driven and ‐overexpression lines were found to show enhanced chilling tolerance, indicating that the introduction of *RGA4L* can rescue the attenuated chilling tolerance of NIP‐NIL‐*qCSS12* ^*ind*^ (Figures 2k–n and S2j,k). Moreover, NJ11‐, HHZ‐, and 556‐NIL‐*qCSS12* ^*jap*^ exhibited enhanced chilling tolerance (Figure S2l–n). These results indicate that *RGA4L* is the causal gene of *qCSS12* and functions as a positive regulator for chilling tolerance in rice. Furthermore, the recombination event in R32 introduces a segment containing a critical 34‐nucleotide into the CDS of *RGA4L* ^*ind*^, creating an *RGA4L* ^*ind*^‐*RGA4L* ^*jap*^ chimeric gene (Figures 1f and S1b). In combination with the chilling tolerance of R32, we show that the 34‐bp variation in *RGA4L* accounts for chilling tolerance divergence between the two parents. However, we found that *RGA4L* was unable to improve chilling tolerance at the budding stages or improve tolerance to other abiotic stresses, such as heat, oxidative stress, salinity and drought (Figure S3a–j).

### 2.3 *RGA4L* Encodes a New Type NLR Protein

**FIGURE 3:**
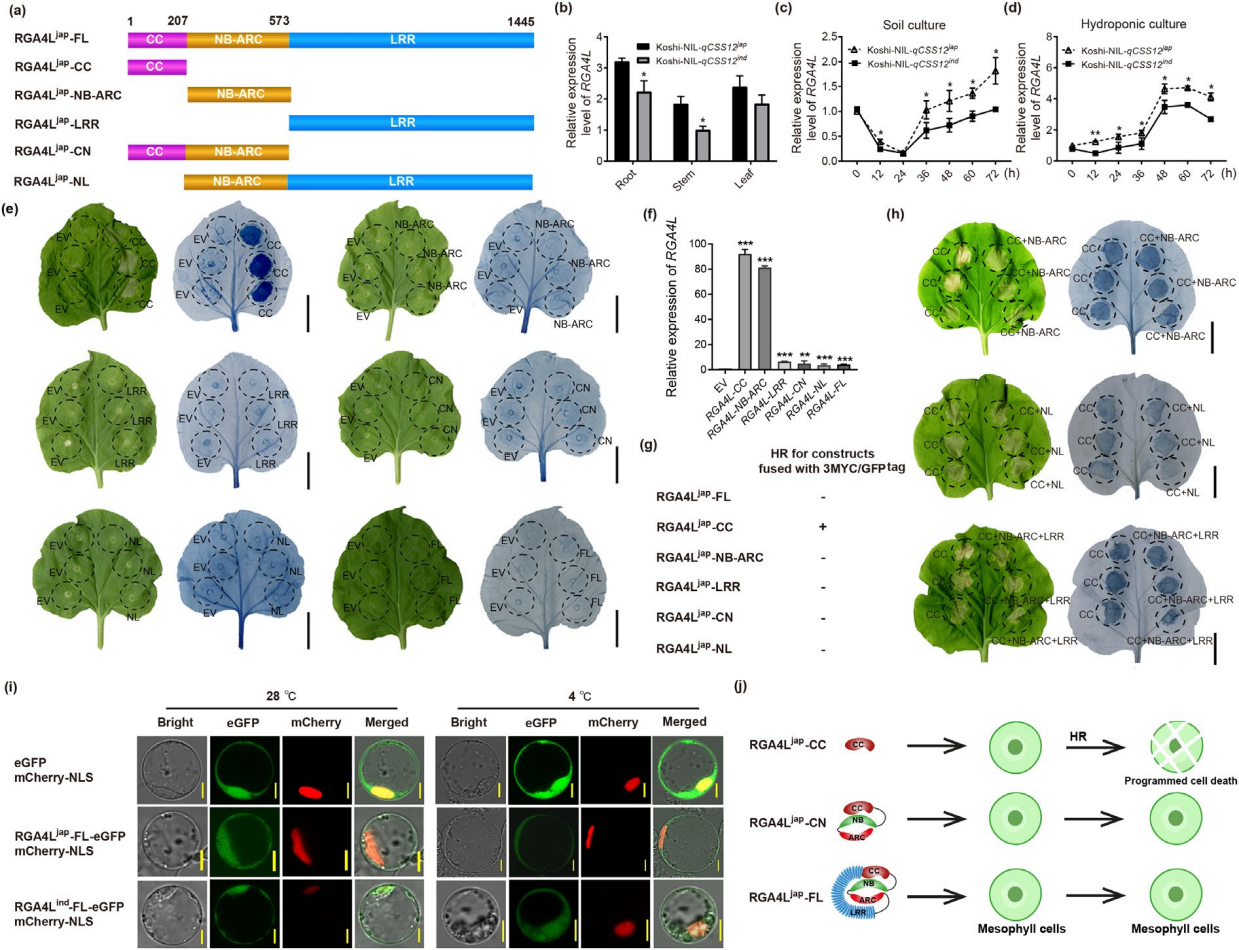
Functional characterisation of *RGA4L* gene. (a) Schematic showing plasmids containing the domains of RGA4L^jap^. The positions of the amino acids defining domain boundaries are indicated at the top. (b) Expression of *RGA4L* in roots, stems, and leaves under chilling stress. *Actin* was used as a reference gene. The value from the stem of Koshi‐NIL‐*qCSS12* ^*ind*^ was normalised as 1. (c, d) Chilling stress‐induced expression of *RGA4L* in the leaves of rice seedlings under soil (c) and in hydroponic (d) culture conditions. (e) Trypan blue staining of tobacco leaves at 5 days post infiltration (dpi). Each leaf is infiltrated with the empty vector (EV) on the left side as a negative control, and the right side is infiltrated with the plasmids containing the truncated domains of RGA4L^jap^ protein (15 replicates each). (f) qPCR to test the expression of the full‐length and truncated domains of *RGA4L*. The transcript levels of EV are normalised as ‘1’. (g) HR induction (+) or not (−) for each construction. (h) Trypan blue staining of tobacco leaves. The CC domain infiltrated on the left side was used as a positive control, and the domain combinations of RGA4L^jap^ were infiltrated on the right side (15 replicates each). (i) Subcellular localisation of RGA4L in rice protoplasts under 28°C (left) and 4°C (right). Green fluorescence represents GFP‐fused RGA4L, while red fluorescence represents mCherry‐fused nucleus marker NLS. Empty vector was used as a negative control. (j) A working model for HR induced by functional CC domain. Scale bars in (e) and (h), 1 cm; scale bars in (i), 5 μm. Data are shown as mean ± SD, *n* = 3 in (b–d), *n* = 6 in (f), *p < 0.05, **p < 0.01, ***p < 0.001. ARC, abbreviated from Apaf‐1, R proteins and CED‐4; CC, coiled‐coil; CN, contains CC and NB‐ARC domains; FL, full‐length; LRR, leucine‐rich‐repeat; NB, nucleotide binding; NL, contains NB‐ARC and LRR domains.

*RGA4L* is annotated to encode an NLR protein of 1445 amino acids (Figures 3a and S4a). Phylogenetic analysis revealed that RGA4L was grouped with its orthologs of the monocotyledons (Figure S4b). qPCR analysis showed that *RGA4L* is ubiquitously expressed in the roots, stems, and leaves of rice plants (Figure 3b). Furthermore, the expression of *RGA4L* exhibited a dynamic fluctuation in response to chilling stress, with an initial downregulation in the early stages (0–24 h) followed by upregulation at the latter time points (Figure 3c,d). Intriguingly, a significant difference in *RGA4L* expression was observed between Koshi‐NIL‐*qCSS12* ^*jap*^ and ‐*qCSS12* ^*ind*^ during chilling stress (Figure 3c,d). However, no significant difference in *DREB1A*, *B* and *C* expression was detected in the two genotypes under chilling stress (Figure S5a).

In general, NLR genes can induce hypersensitive response (HR)‐associated cell death, restricting pathogen infection and conferring disease resistance (Wang, Zhang, et al. 2015). To test the HR activity of *RGA4L*, we infiltrated tobacco leaves with the *Agrobacterium* strain GV3101 carrying plasmids containing the *CaMV* 35S promoter‐driven full‐length CDS or domain truncated fragments of *RGA4L* ^*jap*^. Here, we encountered difficulty in detecting full‐length and long truncated forms (NB, CN, NL, and LRR) of RGA4L protein in the leaf samples due to extremely low expressions (Figure S5d,e), and therefore performed qPCR to determine expression level. Significantly higher expression was detected of either the full‐length or truncated domains of *RGA4L* ^*jap*^ compared to the empty vector control (Figure 3f). We found that HR phenotypes were observed only in the areas with CC domain infiltration (Figure 3e,g,h). Similar results were also observed with the corresponding *RGA4L* ^*ind*^ constructs (Figure S5b,c,f). This indicated that the CC domain solely, but not others, induces HR. The NB‐ARC domain is proposed to function as a molecular switch, regulating NLR protein activity through intra‐molecular interactions with the CC and LRR domains (Takken and Goverse 2012; Wang, Ji, et al. 2015). Given that CC, but not the other domains or full length of RGA4L, induces HR, we wondered if the intra‐molecular interaction exists in RGA4L, which could inhibit CC‐induced HR. Hence, we further examined these intra‐interactions among the CC, NB‐ARC, and LRR domains in tobacco leaves. In line with previous reports, HR was observed when co‐expression of *RGA4L* ^*jap*^ CC with either the NB‐ARC, NB‐LRR (NL), or LRR domain *in trans*, but not *in cis* (Figure 3e,h and Table S3), suggesting that LRR might interact with NB‐ARC to inhibit CC‐induced HR *in cis* (Figure 3j). Collectively, these results indicate that *RGA4L* encodes a protein with HR‐inducing activity.

To examine the subcellular localisation of RGA4L, we performed transient expression analysis in rice protoplasts. The results showed that both the full‐length and truncated forms of RGA4L^jap^ and RGA4L^ind^ protein were localised to both the cytoplasm and nucleus (Figures 3i and S6). Considering the nucleocytoplasmic localisation of RGA4L, we wondered if chilling alters this distribution. Chilling of the rice protoplasts transiently expressing RGA4L did not alter the localisation of either full‐length or truncated RGA4L^jap^ (Figures 3i and S6).

NLR genes play crucial roles in perceiving pathogens and triggering plant defence responses (Liu et al. 2015; Wang, Zhang, et al. 2015). However, when Koshi‐NIL‐*qCSS12* ^*jap*^ or ‐*qCSS12* ^*ind*^ were infested with planthoppers (BPH; *Nilaparvata lugens Stål*) for 14 days, both genotypes exhibited a similar high susceptibility to BPH (Figure S3k,l). Further, respective inoculation of the two genotypes with *X. oryzae pv. oryzicola*, *X. oryzae pv. oryzae*, and *Rhizoctonia solani* did not result in significantly different lesion lengths (Figure S3m–r). These results suggest that *RGA4L* is a new type of NLR gene involved in chilling tolerance in *japonica* rice, but not in plant defence responses.

### 2.4 RGA4L Interacts With OsHSP90 and OsLEA5 to Regulate Chilling Tolerance in Rice

**FIGURE 4:**
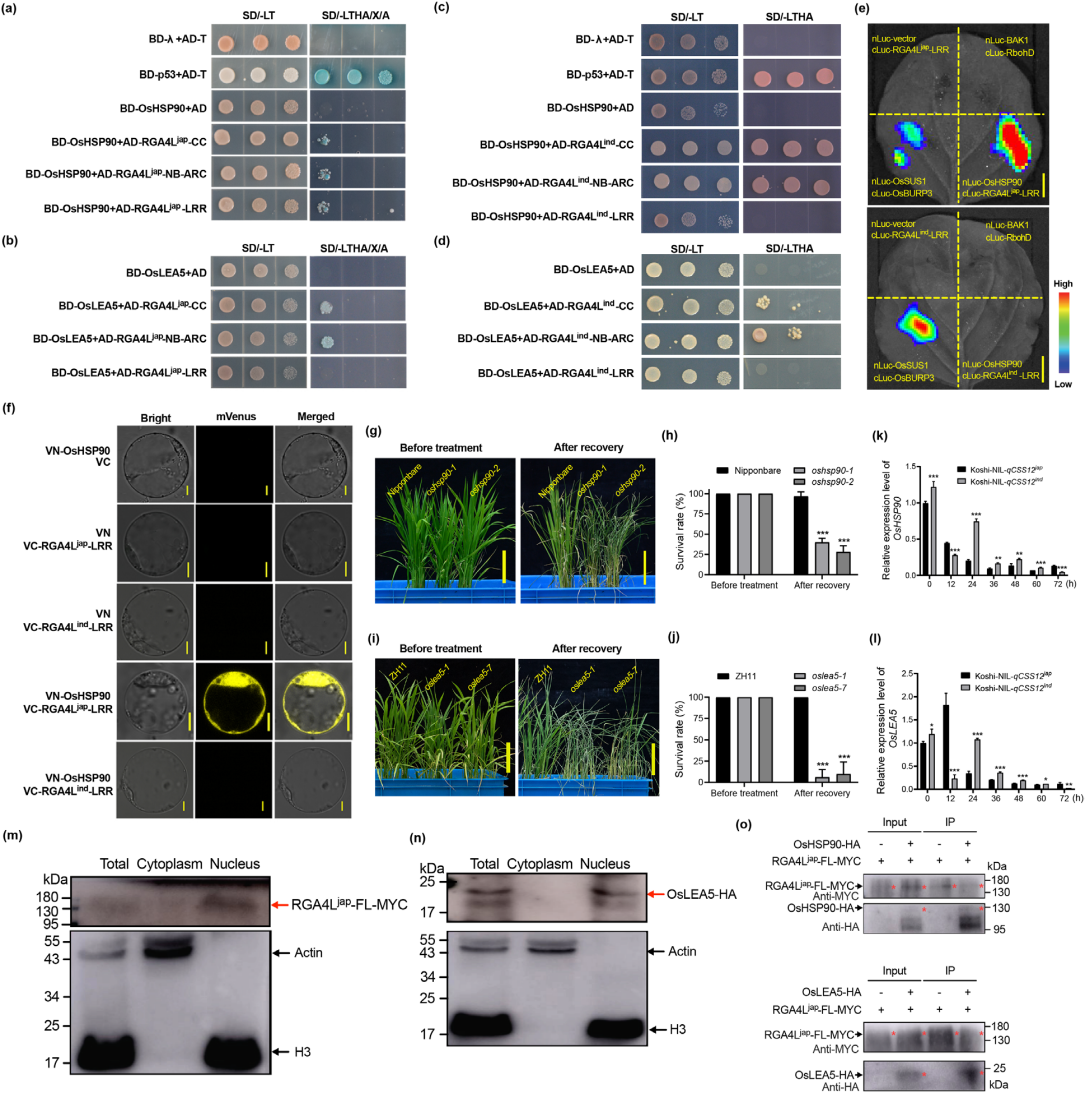
OsHSP90 and OsLEA5 physically interact with RGA4L and are involved in chilling tolerance. (a, b) Interaction assay of RGA4L^jap^ and OsHSP90 (a)/OsLEA5 (b) by Y2H. (c, d) Interaction assay of RGA4L^ind^ and OsHSP90 (c)/OsLEA5 (d) by Y2H. BD‐λ + AD‐T was used as a negative control, while BD‐p53 + AD‐T was used as a positive control. (e) Interaction assay of both RGA4L^jap^ and RGA4L^ind^‐LRR with OsHSP90 by LCA in tobacco leaves. nLuc‐BAK1/cLuc‐RbohD and nLuc‐OsSUS1/cLuc‐OsBURP3 were used as the negative and positive controls, respectively. (f) Interaction assay of both RGA4L^jap^ and RGA4L^ind^‐LRR with OsHSP90 by BiFC in rice protoplasts. Empty vectors containing the N‐terminus or C‐terminus of mVenus (mVN or mVC) were used as the negative controls. (g–j) Chilling stress phenotypes and survival rates of wild‐type and *OsHSP90* (g, h)/*OsLEA5* (i, j) mutant seedlings. (k, l) Expression of *OsHSP90* (k) and *OsLEA5* (l) in leaves under chilling stress. *Actin* was used as a reference gene. The value before chilling treatment (0 h) of Koshi‐NIL‐*qCSS12* ^*jap*^ was normalised as 1. (m, n) Detection of RGA4L (m) and OsLEA5 (n) in the total, cytoplasm, and nucleus fractions of rice protoplasts. RGA4L and OsLEA5 proteins were detected by anti‐MYC antibody. Anti‐Tubulin and anti‐H3 antibodies were used as internal controls. (o) Co‐IP assays showing the interactions between RGA4L (MYC‐tag) and OsHSP90/OsLEA5 (HA‐tag). Proteins were immunoprecipitated by anti‐MYC and anti‐HA antibodies. Scale bars in (e), 1 cm; scale bars in (f), 5 μm; scale bars in (g) and (i), 5 cm. Data are shown as mean ± SD, *n* = 3, *p < 0.05, **p < 0.01, ***p < 0.001. A, Aureobasidin A; SD/−LT, synthetic dropout without Leu and Trp; SD/−LTHA, synthetic dropout without Leu, Trp, His, and Ade; X, X‐α‐Gal.

To gain deeper insight into the molecular mechanism of *RGA4L*, we first predicted the structure of RGA4L^jap^ and RGA4L^ind^ proteins using ColabFold based on AlphaFold2 (Mirdita et al. 2022). By and large, no difference was observed in the global structures of the two proteins (Figure S7a,b). However, surprisingly, a notable difference was detected in their C‐terminal regions. The 34‐bp deletion at the 3′‐end of *RGA4L* ^*ind*^ leads to a substantial change in its secondary structure (Figure S7a,b). Specifically, the C‐terminus of RGA4L^jap^ forms a flexible random coil, whereas that of RGA4L^ind^ forms an alpha‐helix structure (Figure S7a,b). This structural difference may underlie the functional divergence of the two alleles. Subsequently, we identified interacting proteins of RGA4L through yeast two‐hybrid cDNA library screening using the truncated‐CC, ‐NB‐ARC, and ‐LRR domains of RGA4L^jap^ as baits. Nineteen candidates were identified (Table S4), with a particular focus on *OsHSP90* (*LOC_Os06g50300*) and *OsLEA5* (*LOC_Os05g50710*). OsHSP90, a molecular chaperone essential for the stability of NLR proteins (Liu et al. 2015; Shirasu 2009; Thao et al. 2007; Wang et al. 2008), was found to interact respectively with the CC, NB‐ARC, and LRR domains of RGA4L^jap^ in yeast. However, we could not detect the interaction of OsHSP90 with the LRR domain of RGA4L^ind^ (Figure 4a,c). OsLEA5, a protein known to confer abiotic stress tolerance in rice (Huang, Jia, et al. 2018; Huang, Zhang, et al. 2018), was detected to interact with either these truncated proteins of RGA4L^jap^ or RGA4L^ind^, with the exception of the LRR domain (Figure 4b,d). These interactions were further validated in vivo and in vitro through luciferase complementation assays (LCA), bimolecular fluorescence (BiFC), and pull‐down assays (Figures 4e,f and S8a–h). The lack of interaction between the RGA4L^ind^ LRR domain and OsHSP90 could be attributed to its 34‐bp deletion (Figure 1c,e,f).

To understand the functions of *OsHSP90* and *OsLEA5* in *RGA4L*‐mediated chilling stress tolerance, we disrupted the two genes in Nipponbare and ZH11 (a chilling tolerant variety) respectively, through CRISPR/Cas9 technology (Figure S2e,f). Mutants of *OsHSP90* and *OsLEA5* exhibited a significantly lower survival rate than respective wild‐type controls (Figure 4g–j). Furthermore, qPCR analyses showed that both *OsHSP90* and *OsLEA5*, whose expression patterns are similar to that of *RGA4L*, were induced in response to chilling stress (Figure 4k,l). Further, co‐localisation and cell fractionation analyses showed that OsHSP90 and RGA4L predominantly co‐localise in the ER (Figure S9b), while OsLEA5 and RGA4L primarily co‐localise in the nucleus (Figures 3i, 4m,n and S9a). Likewise, low temperature did not change their localisations (Figure S9a,b). To determine the subcellular compartmentalisation of RGA4L interaction patterns**—**with OsHSP90 in the ER for possible post‐translational modifications, and with OsLEA5 in the nucleus for its regulatory functions, we performed a semi‐in vivo Co‐IP assay due to difficulty in co‐expressing full‐length *RGA4L* alongside these interaction partners in rice protoplasts. The results confirmed that full‐length RGA4L interacts with both OsHSP90 and OsLEA5. Taken together with the observed co‐localisation patterns, these results provided indirect evidence for subcellular‐compartmentalised interactions of RGA4L. Therefore, we speculate that the interaction between OsHSP90 and RGA4L appears required for the proper assembly of the RGA4L protein complex in the ER, and the complex may be translocated to the nucleus to trigger *OsLEA5*‐mediated downstream regulatory pathways. These results collectively indicate that RGA4L interacts with OsHSP90 and OsLEA5 to confer chilling tolerance.

### 2.5 *RGA4L* Is a Highly Promising Gene for Rice Breeding

**FIGURE 5:**
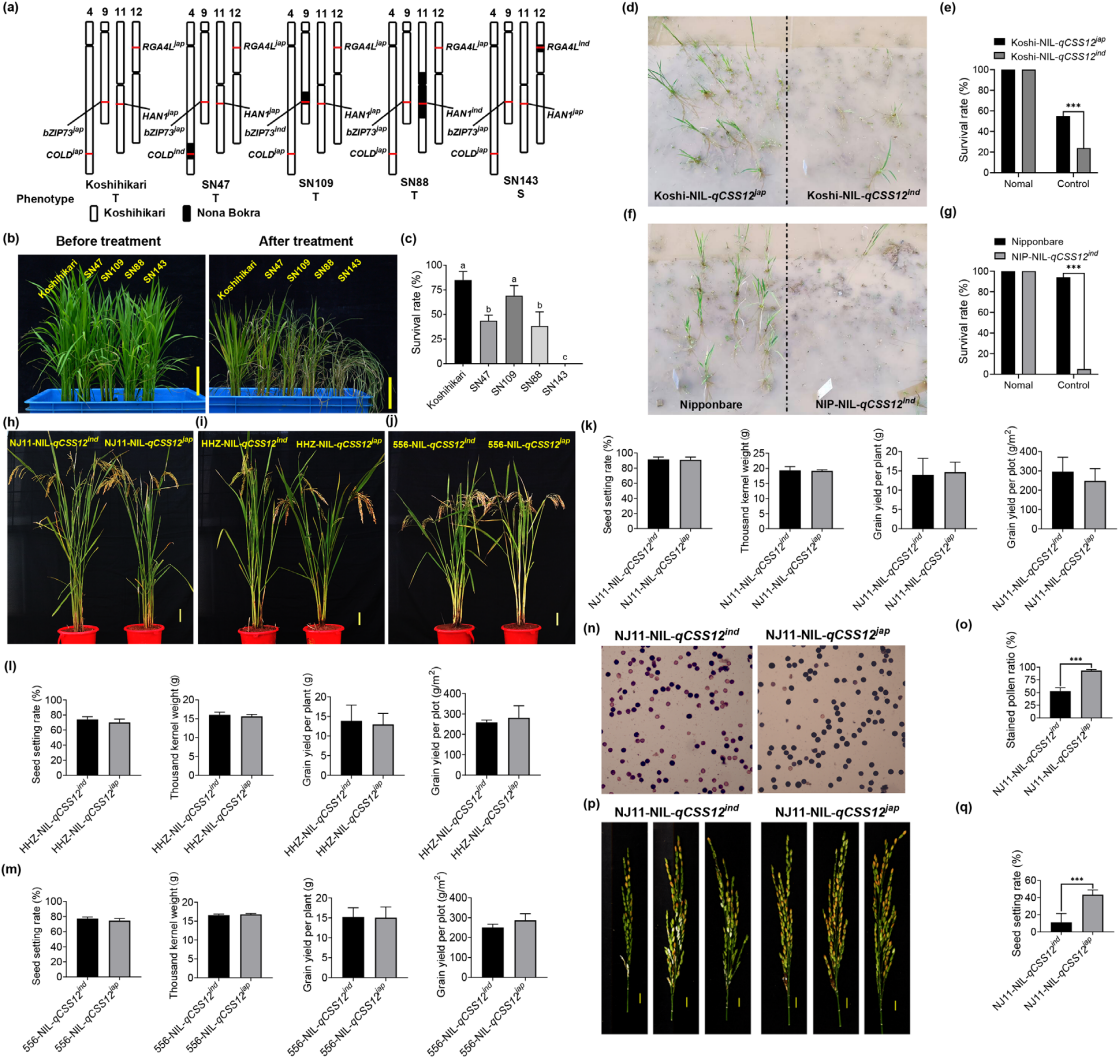
*RGA4L* confers strong chilling tolerance without yield penalty in rice. (a) Genotype schematic of *RGA4L* and other cold tolerance‐related genes in Koshihikari, Nona Bokra and CSSLs. The white and black bars represent the homozygous regions of Koshihikari and Nona Bokra, respectively. The red lines indicate the positions of the cold tolerance genes. S, chilling sensitive; T, chilling tolerant. (b, c) Chilling stress phenotypes (b) and survival rate (c) of Koshihikari, Nona Bokra, and CSSLs seedlings. (d–g) Phenotypes (d, f) and survival rate (e, g) of the *japonica* background NILs seedlings under natural cold conditions in the winter. (h–m) Phenotypes (h–j) and statistical results (k–m) of the yield‐related traits of the *indica* background NILs at the maturation stage. (n, o) Evaluation of pollen fertilities by I_2_–KI staining (n), and comparison of the pollen fertilities (o) of NJ11‐NIL‐*qCSS12* ^*ind*^ and ‐*qCSS12* ^*jap*^ under chilling stress. (p, q) Panicles (p) and seed setting rate (q) of NJ11‐NIL‐*qCSS12* ^*ind*^ and ‐*qCSS12* ^*jap*^ under chilling stress. Scale bars in (b), 5 cm; scale bars in (h–j), 10 cm; scale bars in (p), 1 cm. Data are shown as mean ± SD, *n* = 3 in (c, e, g), and grain yield per plot (g/m^2^) in (k–m), *n* = 10 for the rest in (k–m), *n* = 5 in (o, q). ***p < 0.001.

The above results indicate that *RGA4L* is a novel chilling tolerance gene in rice. To understand its relationship to other well characterised genes in regulating chilling tolerance, we analysed the genetic effect of *RGA4L* on *COLD1* (Ma et al. 2015), *bZIP73* (Liu et al. 2018), and *HAN1* (Mao et al. 2019) using four CSSL lines of SN47, SN88, SN109 and SN143 (Figure 5a). Phenotypic analysis showed that SN143, carrying the chilling‐tolerant *japonica* haplotypes of *COLD1*, *bZIP73* and *HAN1* and an *indica* haplotype *RGA4L*, exhibits a chilling‐sensitive phenotype. In contrast, SN47, SN88 and SN109, each containing the *japonica* haplotype of *RGA4L*, all exhibit a strong chilling tolerance phenotype (Figure 5a–c), suggesting a major epistatic effect of *RGA4L* on these genes. A combined haplotype analysis also confirmed the effect of *RGA4L* on these genes, including *CTB4a* (Zhang et al. 2017) (Figure S10). Further, paddy field trials with the NILs carrying *qCSS12* ^*jap*^ were conducted in the winter of 2023 with an average minimum temperature of 6°C in Guangxi province. These trials showed a significantly higher survival rate in NILs containing *qCSS12* ^*jap*^ than those with the *qCSS12* ^*ind*^ locus (Figure 5d–g), demonstrating the critical role of *RGA4L* in rice chilling tolerance.

To explore the impact of *RGA4L* on the agronomic traits of *indica* rice, we performed field plot trials and investigated the performance of the *indica* background NIL materials in two sites: Nanning, Guangxi province, and Danzhou, Hainan province. This showed that the introduction of *RGA4L* ^*jap*^ did not have a significant detrimental effect on the agronomic traits of *indica* rice (Figures 5h–m and S11). Interestingly, the introduction of *qCSS12* ^*jap*^ into NJ11 (NJ11‐NIL‐*qCSS12* ^*jap*^) can also mitigate yield loss of the rice under chilling stress (Figure 5n–q). These data collectively indicate that *RGA4L* is a valuable gene for improving the chilling tolerance of rice.

### 2.6 *RGA4L* Is a Target of Selection for Chilling Tolerance During the Domestication of *Japonica* Rice

In general, *japonica* rice is more tolerant to chilling stress compared to *indica*. To confirm the association of *RGA4L* with the differences in chilling tolerance among rice subspecies, we used a molecular marker, QS, which was designed based on its 34‐bp indel variation, to examine the genotypes of *RGA4L* in 136 rice varieties (Table S5). We found that 88.89% of the tropical *japonica* and 96.55% of the temperate *japonica* rice carry the *RGA4L* ^*jap*^ homozygous genotype, while 76.06% of the *indica* rice carries the *RGA4L* ^*ind*^ homozygous genotype (Table S6). Intriguingly, the vast majority of tropical *japonica* (93.75%) and temperate *japonica* (96.25%) varieties containing homozygous *RGA4L* ^*jap*^ exhibit a higher chilling tolerance, with a survival rate exceeding 50%, whereas 100% of *indica* varieties containing homozygous *RGA4L* ^*ind*^ showed a chilling‐sensitive phenotype with a survival rate below 50% (Table S6), indicative of a high correlation between *RGA4L* and chilling tolerance.

**FIGURE 6:**
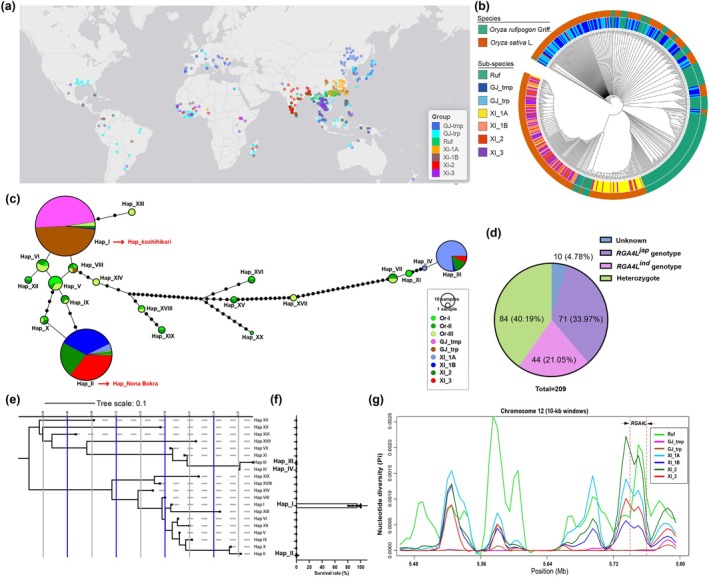
The geographic and phylogenetic origins of *RGA4L*. (a) Geographic distributions among 515 accessions. Coloured circles represent different rice subspecies. (b) Phylogenetic tree of *RGA4L*. (c) Haplotype network of *RGA4L*. Circle size is proportional to the number of samples for a given haplotype. Black spots represent unobserved, but inferred haplotypes. Lines between haplotypes represent mutational steps between alleles. The two red arrows indicate the haplotypes of Koshihikari and Nona Bokra, respectively. (d) Frequency of *RGA4L* alleles identified in common wild rice. (e) Phylogenetic diagram showing evolutionary relationships between the haplotypes of *RGA4L*. (f) Survival rate of rice varieties with a certain haplotype after chilling treatment. Data are shown as the mean ± SD, *n* = 3. (g) Nucleotide diversity of *RGA4L* in the cultivar populations and the wild rice group ( *Oryza rufipogon* Griff.). The *x*‐axis denotes the position of *RGA4L* and its flanking regions on chromosome 12 and the *y*‐axis indicates average *π* values. The red dotted lines indicate the location of *RGA4L*. GJ_tmp, temperate *japonica* rice; GJ_trp, tropical *japonica* rice; Ruf, *Oryza rufipogon* Griff.; XI, *xian_indica* rice.

To further investigate whether *RGA4L* has undergone artificial selection, we analysed the nucleotide diversity of *RGA4L* using 515 representative accessions, comprising 172 wild accessions and 343 varieties that were selected from 3K rice (Wang et al. 2018) (Table S7). The selected accessions were widely distributed, spanning tropical to temperate regions, especially in East and Southeast Asia (Figure 6a). Phylogenetic analysis showed that *RGA4L* is quite diverse across the range of wild rice, *indica*, and *japonica* accessions (Figure 6b). We used nucleotide polymorphisms of *RGA4L* to group these accessions into 20 major haplotypes (Figure 6c). Among them, Hap_I is predominantly found in *japonica* rice, including Koshihikari; Hap_II, mainly representing the Nona Bokra haplotype; and Hap_III and Hap_IV are primarily present in the *indica* accessions (Figure 6c). Our findings suggest that *RGA4L* has undergone differentiation in populations of common wild rice ( *O. rufipogon* Griff.) (Figure 6c,e). In line with this, genotyping revealed that 33.97% of Guangxi common wild rice carries the *RGA4L* ^*jap*^ genotype and 21.05% carries the *RGA4L* ^ind^ genotype, while 40.19% of them are heterozygotes (Figure 6d; Table S8). Phenotypic analysis showed that rice with Hap_I exhibits a higher chilling tolerance than those with Hap_II, Hap_III or Hap_IV haplotypes (Figure 6f). These results suggest that both Hap_I (Hap‐Koshihikari) and Hap_II (Hap‐Nona Bokra) directly originated from common wild rice during rice domestication (Figure 6c).

The predominant presence of Hap_I in *japonica* rice suggests this haplotype was selected during domestication. To explore this hypothesis, we compared *RGA4L* nucleotide diversity among *O. rufipogon* , *indica* (XI_1A, XI_1B, XI_2 and XI_3), and *japonica* (GJ_tmp and GJ_trp) rice. On average, *japonica* exhibits a lower diversity (*π* = 0.0006, *θ* = 0.0042) than that of *O. rufipogon* (*π* = 0.1410, *θ* = 0.1501) and *indica* (*π* = 0.1146, *θ* = 0.1193) (Figure 6g; Table S9). Additionally, a significant negative Tajima's *D* (Tajima 1989) was observed in *japonica* rice (Tables S9 and S10), indicating a deviation from neutral evolution. The decreased nucleotide diversity could result from either selective pressures or demographic history, such as a bottleneck effect during domestication (Oleksyk et al. 2010). To distinguish these two effects, we further analysed nucleotide diversity in the 250‐kb flanking regions of *RGA4L*. The average diversity of the flanking regions (upstream: *π* = 0.0018, *θ* = 0.0037; downstream: *π* = 0.0139, *θ* = 0.0062) was much higher than in *RGA4L* (*π* = 0.0002, *θ* = 0.0013) in the GJ_tmp subgroup (mainly containing Hap I) (Table S9). Considering that selection could result in changes in the nucleotide diversity of the locus, the decrease in nucleotide diversity at the *RGA4L* locus in GJ_tmp may be largely due to positive selection. To support this, we further performed an MLHKA test (Wright and Charlesworth 2004) on *RGA4L* sequences of four taxa in reference to six neutral genes (Zhu et al. 2007) and found a significant *P*‐value for *japonica* rice (*p* = 0.0028) (Table S10), indicating a strong artificial selection happened on *RGA4L* during *japonica* domestication. Taken together, these findings suggest that natural variation in *RGA4L*, specifically the 34‐bp insertion, might be one of the ancestral alleles retained in populations of *O. rufipogon* and had undergone artificial selection to enhance chilling tolerance during the domestication of *japonica* rice.

## 3 Discussion

Isolation of novel genes underlying chilling stress tolerance is of critical importance for breeding chilling‐tolerant rice cultivars. In this study, we isolated a novel major chilling‐tolerant QTL, *qCSS12*, through a map‐based cloning approach in rice and functionally characterised its causal gene *RGA4L*, which encodes a specific NLR protein (Figures 1 and S1). The NLR protein family is so far known to be involved only in perceiving pathogens and triggering plant defence responses (Du et al. 2021; Liu et al. 2015; Wang, Zhang, et al. 2015). Previous studies have demonstrated that NLR proteins detect pathogen effectors by either direct or indirect effector‐triggered immunity (ETI), thereby leading to a hypersensitive response and a disease‐resistance phenotype (Cesari et al. 2014; Wang, Zhang, et al. 2015). However, although the *RGA4L* CC domain alone or CC plus other domains *in trans* can induce HR in tobacco leaves (Figures 3e,g,h and S5c,f; Table S3), we surprisingly found no evidence that *RGA4L* is involved in insect and disease resistance (Figure S3k–r). These data identify a novel NLR gene that regulates chilling tolerance in rice, although the functions of NLR genes in the abiotic stress response of *Arabidopsis* have been documented in several reports (Ariga et al. 2017; Chini et al. 2004; Huang et al. 2010; Liu et al. 2015; Yang et al. 2010). Our results suggest that higher plants have evolved a mechanism whereby NLR genes can regulate abiotic stresses or modulate the trade‐off between biotic and abiotic stress responses. However, we still could not clarify whether the *RGA4L*‐mediated programmed cell death is correlated with chilling tolerance enhancement in rice in this study.

In general, the auto‐inhibited state of NLR proteins is maintained by molecular chaperones, such as OsHSP90, and its co‐chaperones OsRAR1 and OsSGT1, which are essential for proper assembly of NB‐LRR proteins (Takken and Goverse 2012; Thao et al. 2007; Wang et al. 2008). In our study, the LRR domain of RGA4L^jap^ could physically interact with OsHSP90 (Figure 4a,e,f), whereas that of RGA4L^ind^ with a 34‐bp deletion at the 3′‐end of its CDS was unable to interact with OsHSP90 (Figure 4c,e,f). This suggests that the interaction of the RGA4L LRR domain with OsHSP90 may be essential for properly assembling the RGA4L protein, facilitating an auto‐inhibited conformation in the absence of stress signals (Heidrich et al. 2012). Due to technical difficulties in producing enough RGA4L protein in prokaryotic and eukaryotic expression systems, we still lack solid biochemical evidence to support the above statement. Moreover, several further questions remain unanswered in this study, including how rice plants perceive chilling signals, whether *RGA4L* executes its function similar to known NLR proteins, and how *RGA4L* transduces the chilling signal to *OsLEA5*‐mediated downstream pathways. These questions are worthy of further analysis.

The predominant haplotype of *RGA4L* (Hap I) confers chilling tolerance, whereas the Hap II haplotype of *RGA4L* in Nona Bokra, which is mainly present in *indica*, confers a chilling‐sensitive phenotype (Figure 6c,f). Nucleotide diversity analysis indicated a strong artificial selection effect on the *RGA4L* locus during *japonica* rice domestication (Figure 6g; Tables S9 and S10). Regarding Hap I and Hap II, two predominant haplotypes of *japonica* and *indica* rice, respectively, were also identified in rice progenitor *O. rufipogon* , in which the *Or‐III* group contains Hap I, and the *Or‐I* group contains Hap II (Figure 6c). It has been proposed that *Or‐III* is the progenitor of *japonica*, and *Or‐I* is the progenitor of *indica* rice (Huang et al. 2012). This suggests that *O. rufipogon* has undergone functional variation in *RGA4L* in the form of a 34‐bp Indel. In agreement with this, molecular marker analysis revealed that the 34‐bp mutation in *RGA4L* is ubiquitously found in *O. rufipogon* accessions, either in the homozygous or heterozygous state (Figure 6d).

**FIGURE 7:**
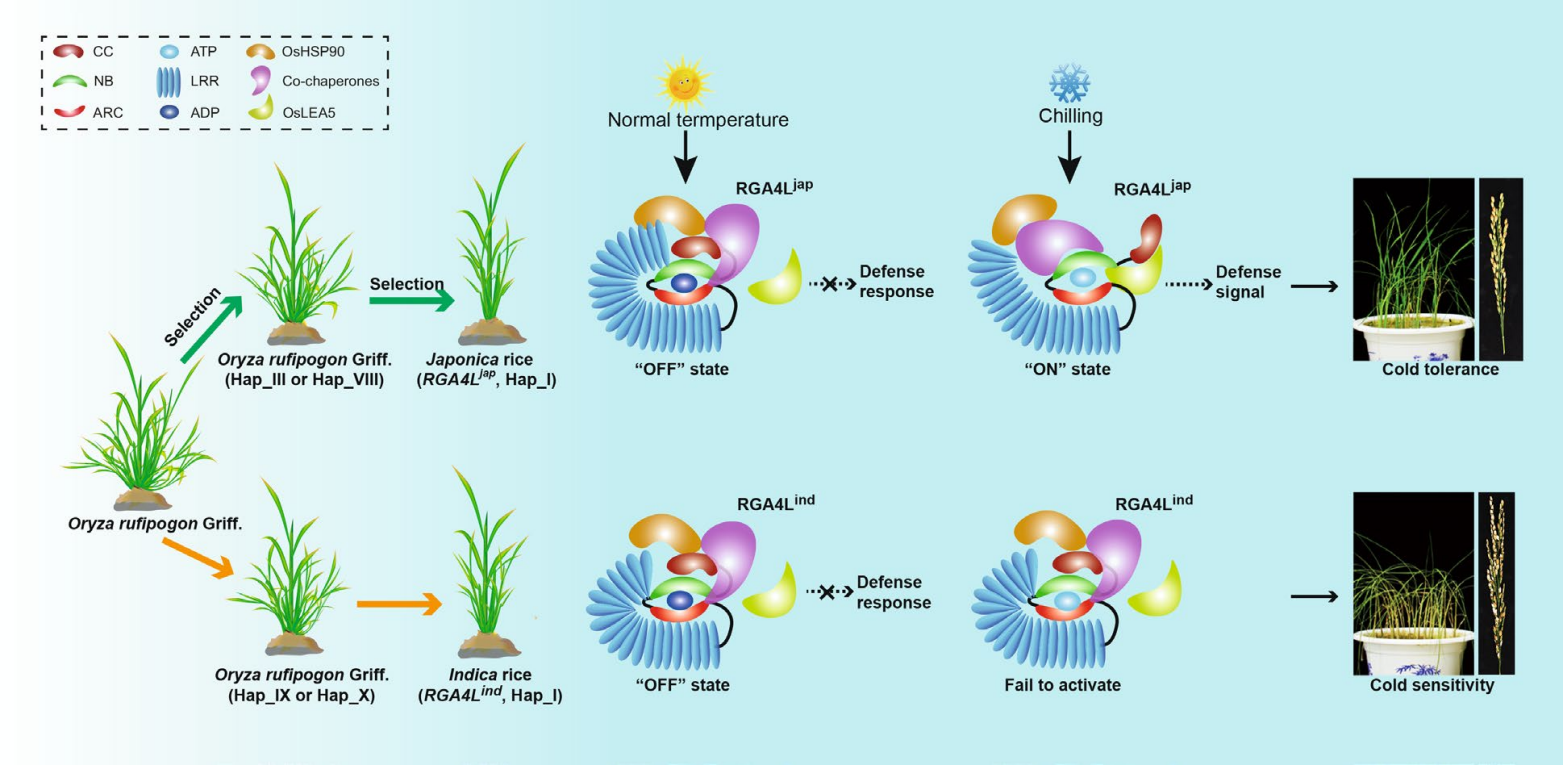
A working model was proposed to depict the selection of *RGA4L* and its molecular mechanism underlying chilling tolerance. *RGA4L* ^*jap*^ was a target of artificial selection during *japonica* domestication, facilitating the cultivation of *japonica* rice cultivated in temperate or high‐altitude regions. OsHSP90 and its co‐chaperones can physically interact with the LRR domain of RGA4L^jap^, enabling the proper assembly of the protein complexes to switch between resting and activated states of RGA4L. This interaction may allow for the perception and transduction of chilling signals, thereby triggering *OsLEA5*‐regulated downstream pathways and conferring a chilling tolerant phenotype in rice.

Here, we proposed a working model to illustrate the inferred history of *RGA4L*‐targeted *japonica* domestication and the potential mechanism underlying *RGA4L*‐mediated chilling tolerance in rice (Figure 7). We speculate that *RGA4L* ^*jap*^, with the 34‐bp insertion, was a target of artificial selection during *japonica* domestication, facilitating the cultivation of *japonica* rice cultivated in temperate or high‐altitude regions. OsHSP90 and its co‐chaperones can physically interact with the LRR domain of RGA4L^jap^, enabling the proper assembly of the protein complexes to switch between resting and activated states of RGA4L. This interaction may allow for the perception and transduction of chilling signals, thereby triggering *OsLEA5*‐regulated downstream pathways and conferring a chilling tolerant phenotype in rice (Figure 7).

As a summary, *RGA4L* is the first NLR gene identified to confer chilling stress tolerance from the seedling to maturation stages in rice. More importantly, *RGA4L* exhibits an epistatic effect on the previously reported chilling tolerance‐related genes, including *COLD1*, *bZIP73*, *CTB4a* and *HAN1* (Liu et al. 2018; Ma et al. 2015; Mao et al. 2019; Zhang et al. 2017), and the introgression of *RGA4L* into the genomes of *indica* rice enhances chilling tolerance without yield penalty. Therefore, *RGA4L* is a highly valuable gene for breeding elite chilling tolerant varieties of rice.

## 4 Materials and Methods

### 4.1 Plant Materials and Growth Conditions

Koshi‐NIL‐*qCSS12* ^*jap*^ and its sib‐lines koshi‐NIL‐*qCSS12* ^*ind*^ were developed from a recombinant containing a heterozygous region delimited by the molecular markers 12M5.673 and 13M5.775 (~102 kb) (Table S1). *Indica* background (NJ11, HHZ and 556) NILs for *qCSS12* ^*jap*^ were generated by crossing the varieties with Koshikikari, and the resulting F_1_ of them were backcrossed with their respective recurrent parents for six generations, respectively. Similarly, the *japonica* background (Nipponbare) NILs for the *qCSS12* ^*ind*^ locus were generated by crossing the varieties with Nona Bokra, and the F_1_ was backcrossed with Nipponbare for six generations. Plant materials were grown following the methods as previously described (Cen et al. 2018). The hydroponic culture was performed as a previously described protocol (Ren et al. 2005). All the field trials were carried out in the experimental paddy located on the campus of Guangxi University, Nanning, Guanxi, and in Danzhou, Hainan province, in compliance with the legislation of China. Tobacco (*N. benthamiana*) was grown at 25°C with a 16 h light/8 h dark photoperiod.

### 4.2 Evaluation of Chilling Tolerance

The rice plants were exposed to a diurnal temperature regime of 8°C/6°C, with a photoperiod of 13 h/11 h, supplemented with 10 000 lx of artificial light and 65% humidity for 7 days. Following the chilling treatment, the rice plants were allowed to recover at 28°C/26°C (day/night) for an additional 7 days. Chilling tolerance was evaluated according to Kim's method (Kim et al. 2012) with minor modifications. In brief, a visual assessment was performed using a rating scale from 1 (highly tolerant) to 9 (highly sensitive), as previously described (Andaya and Mackill 2003), to grade the chilling tolerance. After recovery, the visual rating and survival rate of the chilling‐treated seedlings/plants were recorded accordingly.

### 4.3 Map‐Based Cloning of *RGA4L*

The CSSL SN143 was backcrossed with Koshihikari to construct a BC_4_F_2_ population for the fine mapping of *qCSS12*. First, a small‐scale F_2_ population consisting of 428 individuals was developed to determine the genetic basis of the locus. Genotyping and chilling stress phenotyping of the population were conducted following the methods described above. A chi‐squared test (*χ* ^2^, *p* < 0.05) was performed to assess the segregation ratio of the chilling tolerance phenotype. Subsequently, a larger mapping population of 6813 individuals was then used for further fine mapping. The primer sequences of the molecular markers are provided in Table S1. Homozygous recombinants were identified through molecular markers and their phenotypes were determined through F_3_ progeny testing. The QTL analysis software QTL IciMapping 4.1 (Meng et al. 2015) was utilised to perform the genetic linkage analysis and QTL identification (LOD ≥ 3). The allelic variations of *RGA4L* in Koshihikari and Nona Bokra were determined by sequencing.

### 4.4 RNA Isolation and qPCR Analysis

Total RNA was extracted using an RNAprep Pure Plant Kit (DP441, TIANGEN, China) and reverse transcribed into cDNA with a HiScript 1st Strand cDNA Synthesis Kit (R111, Vazyme, China), following the manufacturer's instructions. qPCR was performed on a Roche LightCycler 480 Real‐Time PCR System in 10 μL reactions using the ChamQ SYBR qPCR Master Mix (Without ROX) (Q321, Vazyme, China). The relative expression of each gene was calculated based on the 2^−ΔΔCT^ method (Livak and Schmittgen 2001). The *Actin* gene (*LOC_Os11g06390*) served as an internal reference.

### 4.5 5′‐Race

The SMARTer RACE 5′/3′ Kit (634 859, Clontech, Japan) was utilised to determine the full‐length cDNA of *RGA4L*. The inner and outer *RGA4L‐*specific primers GSP1 and GSP2 were designed for the differential display of 3′‐end fragments. First‐strand synthesis was conducted following the method described above. PCR products were then cloned into the pEASY vector (CTB111‐01, TransGen, China).

### 4.6 Abiotic Stress Resistance Assays

For heat stress, the rice plants were exposed to a diurnal temperature of 40°C, with a 13‐h day and 11‐h night photoperiod, supplemented with 10 000 lx of artificial light and 65% humidity, for 3 days followed by recovering at 28°C/26°C (day/night) for an additional 7 days. For oxidative stress, H_2_O_2_ was added to the culture medium at concentrations of 10, 20 and 40 mM, respectively, for 3 days. For salt stress, sodium chloride (NaCl) was added to the culture medium at concentrations of 75, 100, 125, 150 and 175 mM, respectively, for 5 days. For drought stress, 25% PEG6000 was added to the culture medium for 10 days, after which the plants were returned to the medium without PEG6000 for 7 days of recovery.

### 4.7 Expression Pattern Analysis

Different tissues (stem, root and leaf) and different time points of the chilling‐treated samples were harvested. Total RNA isolation, RNA reverse transcription, and qPCR were performed following the abovementioned methods. Primer sequences for the relevant genes are listed in Table S1.

### 4.8 Subcellular Localisation and Bimolecular Fluorescence Complementation Assays (BiFC)

The full‐length CDS of *RGA4L* and its interacting genes without stop codons were amplified and cloned into the pA7‐GFP vector, and the resultants were used to determine their subcellular localisation. The pA7‐GFP backbone plasmids were transformed into rice protoplasts through a polyethylene glycol (PEG)‐mediated transient expression system (Chen et al. 2006). The fluorescence signal was observed using a laser confocal microscope (LEICA‐TCS‐SP8MP, Germany). To perform the BiFC assays, the CDS of *RGA4L* and its interacting genes were cloned into the pUC19‐nYFP and pUC19‐cYFP vectors, respectively. The resulting plasmids were then transformed into the rice protoplasts and observed with a confocal laser microscope.

### 4.9 Vector Construction and Genetic Transformation

To knock down the expression of *RGA4L* ^*jap*^, binary vector pTCK303 was used to construct the plasmid containing a 452‐bp fragment of *RGA4L* ^*jap*^ from Koshihikari. For overexpression, the full‐length CDS of *RGA4L* ^*jap*^ was inserted into the pCAMBIA1301‐3Flag vector. To construct a plasmid for the complementation test, a fragment containing a 2000‐bp promoter region, 4338‐bp CDS, and 262‐bp 3′ untranslated region (UTR) of *RGA4L* ^*jap*^ was amplified from Koshihikari and cloned into the vector pCAMBIA1301‐FN with a 3 × Flag tag. To generate the knockout lines for *OsHSP90* and *OsLEA5*, gRNA sequences were selected using the CRISPR‐P online platform (http://cbi.hzau.edu.cn/CRISPR2/). The gRNA sequence was then cloned into the CRISPR/Cas9 vector pYLCRISPR/Cas9‐MH at *BsaI* sites (Ma and Liu 2016). All plasmids were transformed into different rice cultivars/lines via the *Agrobacterium*‐mediated transformation. The details of all constructs are listed in Table S2.

### 4.10 Yeast Two‐Hybrid Assay

cDNA library constructed from the chilling‐treated rice seedlings was utilised to screen the interacting proteins of RGA4L using the Make Your Own Mate &Plate Library system (Clontech, 630 490, Japan). The CC, NB‐ARC, and LRR domains of *RGA4L* ^*jap*^ were respectively cloned into the pGBKT7 vector as the baits. Following self‐activation testing, the bait plasmids and prey cDNA library were co‐transformed into the yeast strain Y2HGlod. The transformed cells were then incubated on the SD/−Leu‐Trp/X‐α‐Gal/AbA plates at 30°C for 3–5 days. The colonies that grew on these plates were subsequently transferred to SD/‐His‐Ade‐Leu‐Trp/X‐α‐Gal/AbA plates and incubated at 30°C for an additional 3–5 days. To validate the interactions, the identified interacting proteins were cloned into the pGBKT7 vector and co‐transformed with the pGADT7 containing the corresponding domains of *RGA4L* ^*jap/ind*^ into the Y2HGlod strain, and cultured on the selective medium described above.

### 4.11 Luciferase Complementation Assay (LCA)

LCA was conducted as described previously (Chen et al. 2006). The CDS of truncated domains of *RGA4L* and its interacting genes were cloned respectively into the pRHVc‐nLUC and pRHVc‐cLUC vectors (He et al. 2018). The resulting plasmids were then transformed into the *Agrobacterium* strain GV3101. The *Agrobacterium* cells harbouring the nLUC and cLUC constructs were co‐infiltrated into tobacco leaves. *N. benthamiana* leaves co‐infiltrated with *Agrobacterium* containing cLUC‐OsBURP3 and OsSUS1‐nLUC, which have been reported to interact with each other (Huang et al. 2025), were used as positive controls; cLUC‐RbohD and BAK1‐nLUC were used as negative controls (Zhou et al. 2018). After 2 days of incubation, the leaves were harvested and soaked in a 1 mM luciferin solution. Luminescence was detected using the IVIS Lumina LT system (PerkinElmer, USA).

### 4.12 Pull‐Down Assay

To test the in vitro interaction between *RGA4L* and its interacting genes, pull‐down assays were performed as previously described (Zhang et al. 2017). The CDS of truncated domains of *RGA4L* and its interacting genes were cloned respectively into the following vectors: pGEX‐KG (*RGA4L‐CC*), pGEX‐4T‐1 (*OsHSP90, OsLEA5*), and pMAL‐c2x (*RGA4L‐NB, OsHSP90, OsLEA5*). The resulting plasmids were then transformed into either the *E. coli* strains BL21 (DE3) and Rosetta (DE3). For the pull‐down assay, 5 μg of purified GST/MBP‐fused RGA4L protein or GST/MBP and OsHSP90/OsLEA5‐MBP/GST proteins were incubated in 1 mL of PBS buffer (pH 7.4, 0.1% NP‐40) at 4°C with gentle agitation for 2 h before the addition of 20 μL of Protein A/G PLUS‐Agarose (Santa Cruz, sc‐2003) or Glutathione Sepharose 4B beads (Yeasen, 20507ES10). The beads were then collected by brief centrifugation, washed 5 times with PBS buffer, and re‐suspended in SDS loading buffer. The samples were then subjected to SDS‐PAGE electrophoresis and analysed by Western blotting using the following antibodies: anti‐GST (Lablead, G1001, dilution, 1:1000) and anti‐MBP (ABclonal, AE077, dilution, 1:3000). The original western blot images are provided in Source data.

### 4.13 Cell Fractionation

Cell fractionation was performed as previously described (Huang et al. 2025). Rice protoplasts overexpressing *RGA4L* ^*jap*^‐*MYC* or *OsLEA5*‐*HA* were harvested and ground into a fine powder with liquid nitrogen and then homogenised with lysis buffer (25 mM Tris–HCl [pH = 7.5], 20 mM KCl, 2 mM EDTA, 2.5 mM MgCl_2_, 25% glycerol, 250 mM sucrose, 5 mM DTT and 2 mM PMSF). The homogenate was filtered with a double layer of Miracloth (Calbiochem, Madison, WI, USA). The flow‐through was considered as'total protei'. Next, the flow‐through was centrifuged at 1000× *g* under 4°C for 10 min. The supernatants were transferred to clean tubes and then centrifuged again at 10 000× *g* and 4°C for 10 min, collecting the supernatant as ‘cytoplasm fraction’. The pellets were washed four times with 1 mL of nuclear resuspension buffer (25 mM Tris–HCl [pH = 7.5], 25% glycerol, 2.5 mM MgCl_2_, 0.3% Triton X‐100 and 2 mM PMSF), followed by resuspension in 500 μL of NRB2 (25 mM Tris–HCl [pH = 7.5], 0.25 M sucrose, 10 mM MgCl_2_, 0.5% Triton X‐100, 5 mM *β*‐mercaptoethanol and 2 mM PMSF). The resuspended nucleus was carefully overlaid on the top of NRB3 (25 mM Tris–HCl [pH = 7.5], 1.7 M sucrose, 10 mM MgCl_2_, 0.5% Triton X‐100, 5 mM *β*‐mercaptoethanol and 2 mM PMSF) and centrifuged at 1000× *g* in a swing‐out rotor at 4°C for 10 min. The pellet was collected as ‘nucleus fraction’. It was detected by Western blot using specific Anti‐MYC (M1002, LABLEAD, China) and Anti‐HA (A02040, Abbkine, China) (1:2000). Anti‐actin (A01050, Abbkine, China) (1:3000) and Anti‐histone H3 (AS10710, Agrisera, Sweden) (1:3000) were used as controls.

### 4.14 Semi‐In Vivo Co‐Immunoprecipitation Assay

Total proteins were extracted from rice protoplasts expressing *35S::RGA4L* ^*jap*^‐*MYC*, *35S::OsHSP90‐HA*, or *35S::OsLEA5*‐*HA* constructs and immunoblotted respectively with anti‐MYC and anti‐HA antibodies to determine their expressions. The total proteins from rice protoplasts expressing *RGA4L* ^*jap*^‐*MYC* were co‐incubated with the ones from the protoplasts expressing *OsHSP90*‐*HA* or *OsLEA5*‐*HA* at 4°C for 3 h, respectively. RGA4L proteins were then immunoprecipitated with anti‐MYC antibody (P02333, LABLEAD, China)/protein A/G beads (SC‐2003; Santa Cruz Biotech., USA). The bound proteins were eluted from the affinity beads by boiling for 5 min in 40 μL 2× SDS loading buffer and analysed by immunoblotting.

### 4.15 Measurement of Electrolyte Leakage

Electrolyte leakage was measured following a previously reported method (Jia et al. 2022). Chilling‐treated leaves of rice plants were collected at various time points, cut into uniform segments, and submerged into tubes with 20 mL double‐distilled water. The tubes were then shaken for 1 h. Initial conductivities of the blank control (A0) and the tubes with leaf segments (A1) were recorded using a portable conductivity meter (AZ86031, China). Subsequently, the tubes were boiled for 15 min and allowed to cool to room temperature, after which the conductivities (A2) were measured. Relative electrolyte leakage was calculated using the formula: A1−A0 to A2−A0. Three biological replicates were performed for each sample.

### 4.16 Biotic Stress Resistance Assays in Rice and HR Assays

To investigate the roles of *RGA4L* in disease/pest resistance, we inoculated Koshi‐NIL‐*qCSS12* ^*jap*^/‐*qCSS12* ^*ind*^ with *X. oryzae* pv. *oryzicola* (strain GX01), *X. oryzae* pv. *oryzae* (strain PXO99^A^), and *Rhizoctonia solani* (strain AG‐1 IA), and infested them with planthoppers (BPH; *Nilaparvata lugens Stål*). The bacterial strains GX01 and PXO99^A^ were cultured in NA medium at 28 for 48 h. Bacterial suspensions (OD_600_ = 0.6) were used to inoculate the fully expanded leaves of rice seedlings. The PXO99^A^ strain was inoculated using the leaf‐tip‐clipping method (Kauffman et al. 1973), while the GX01 strain was inoculated following a previously described method (Liao et al. 2020). The AG‐1 IA strain was inoculated following a previously described protocol (Su'udi et al. 2013). The BPH resistance was evaluated using a previously established method (Zhao, Huang, et al. 2016). Disease resistance was assessed based on lesion length.

HR assay was conducted using tobacco leaves. Cell suspensions (OD_600_ = 0.6) of the *Agrobacterium* strain GV3101, harbouring either full‐length CDS or domain truncated forms of the *RGA4L*, were individually infiltrated or co‐infiltrated into tobacco leaves with needleless syringes. Leaves were sampled 5 days after infiltration, and HR lesions were visualised by Trypan Blue staining.

### 4.17 Phylogenetic and Haplotype Analyses, Nucleotide Diversity and Neutrality Tests

SNP data in *RGA4L* and its 250‐kb flanking regions of 515 rice accessions, including 172 *O. rufipogon* accessions, were obtained from the RICE SNP‐SEEK database (https://snp‐seek.irri.org/) (Mansueto et al. 2017; Wang et al. 2018) and the RiceHap3 database (http://server.ncgr.ac.cn/RiceHap3/home.php#) (Huang et al. 2012), respectively. Haplotype analysis was performed using DnaSP V6.0 (Rozas et al. 2017), and the haplotype network was reconstructed by PopART 1.7 (Bandelt et al. 1999). A phylogenetic tree was constructed using the neighbour‐joining method with 1000 bootstrap replications through Phylip 3.697 (Retief 1999) and visualised by the online tool iTOL (https://itol.embl.de/) (Letunic and Bork 2021). Nucleotide diversity and Tajima's *D* test were estimated using the R package PopGenome (Pfeifer et al. 2014). The Maximum Hudson‐Kreitman‐Aguade (MLHKA) (Wright and Charlesworth 2004) test was used to detect deviations from neutrality in *RGA4L* polymorphisms with a set of known neutral genes, including *Adh1*, *GBSSII*, *Ks1*, *Lhs1*, *Waxy* and *TFIIAg‐1* (Zhu et al. 2007), as controls.

## Author Contributions

J.L. and J.Y.L. conceived and designed the studies. P.G., Y.W., H.W., S.L., J.S., X.L., X.M., W.C., and P.J. performed all the lab experiments. P.G., J.L., H.W., and Y.W. carried out the data processing. J.L. and P.G. drafted the manuscript. J.L., J.Y.L., P.G., and R.L. revised the manuscript. All the authors read and approved the final version of the manuscript.

## Conflicts of Interest

The authors declare no conflicts of interest.

## Supporting information


**Figure S1:** Determination of *qCSS12* and the cDNA of its target. (a) Genome‐wide scanning of the QTL loci for chilling sensitive phenotypes of the CSSLs. The *x*‐axis denotes the location of the molecular markers on chromosomes, and the *y*‐axis indicates the LOD Score. (b) Sequence alignment of the crossing‐over region in the *RGA4L* gene. Only the partial sequences flanking the crossing‐over region are shown. The red underline indicates the partial fragment derived from Koshihikari *RGA4L* ^*jap*^ allele, the yellow underline indicates the crossing‐over region, and the blue underline indicates the partial fragment derived from the Nona Bokra *RGA4L* ^*ind*^ allele. QS, SNP1, and SNP2 represent the molecular markers for determining the alleles of *RGA4L* ^*jap*^/*RGA4L* ^*ind*^ in R32. (c) Sequence alignment of OsGSTZ2 protein. I99V and N184I are the two causal variants that accounted for chilling stress phenotype variations conferred by *qCTS12* (Kim et al. 2011). (d) Agarose gel electrophoresis showing RACE PCR amplification products. GSP, gene specific primer; M, marker; S, chilling sensitive; T, chilling tolerant; UPM, universal primer. (e) Predicted and detected structure of *RGA4L* gene. (f) Sequence alignment of RACE amplification products.

**Figure S2:** Construction of the NILs and transgenic lines in this study. (a) NILs of *qCSS12* constructed in the *japonica* rice. The black bars show the homozygous fragments derived from Nona Bokra, and the white bars indicate the homozygous regions of the Koshihikari or Nipponbare. Fragment substitution sites are shown in the blue box. (b) NILs of *qCSS12* constructed in the *indica* rice. The white bars show the homozygous fragments derived from Koshihikari, and the grey bars indicate the homozygous regions of the NJ11, HHZ, or 556. Fragment substitution sites are shown in the blue box. (c, d) Generation of the *RGA4L* transgenic lines. (c) Schematic of the plasmids constructed for generating RNA interference, complementary, and overexpression lines of *RGA4L*. (d) Expression levels of *RGA4L* in transgenic lines and wild type. The expression levels in wild‐types Nipponbare (background of RNA interference lines) and NIP‐NIL‐*qCSS12* ^*ind*^ (background of complementary lines and overexpression lines) are set as ‘1’. Data are shown as mean ± SD, *n* = 6, ***p < 0.001. (e) Knockout of *OsHSP90* by CRISPR/Cas9 editing technology. (f) Knockout of *OsLEA5* by CRISPR/Cas9 editing technology. (g, h) Survival rate (g) and phenotypes (h) of Koshi‐NIL‐*qCSS12* ^*jap*^ and ‐*qCSS12* ^*ind*^ at the tillering stages. (i–k) Electrolyte leakage of the *RGA4L* RNAi lines (i), the *RGA4L* complementary lines (j), and the *RGA4L* overexpressing lines (k). (l–n) Phenotypes and survival rate of *indica* background NILs. Scale bars in (h, l–n), 5 cm. Data are shown as mean ± SD, *n* = 3 in (l, m), ***p < 0.001.

**Figure S3:** Phenotypes of *RGA4L* in response to abiotic and biotic stresses. (a, b) Chilling stress phenotypes (a) and seedling survival rate (b) of Koshi‐NIL‐*qCSS12* ^*jap*^ and ‐*qCSS12* ^*ind*^ at the budding stage. (c–j) Phenotypes and survival rate of Koshi‐NIL‐*qCSS12* ^*jap*^ and ‐*qCSS12* ^*ind*^ seedlings under heat (c, d), oxidative (e, f), salt (g, h), and drought (i, j) stress. (k, l) Phenotypes (k) and survival rate (l) of rice seedlings infested with planthoppers for 14 days. 1 and 5, GXU184, which were used as a resistance control; 2, Koshi‐NIL‐*qCSS12* ^*jap*^; 3, 9311, as a sensitive control; 4, Koshi‐NIL‐*qCSS12* ^*ind*^. (m, n) Phenotypes (m) and lesion length (n) of rice leaves infiltrated with Xoc ( *X. oryzae* pv. *oryzicola*). Nipponbare was used as a sensitive control. (o, p) Phenotypes (o) and lesion length (p) of rice leaves infiltrated with Xoo ( *X. oryzae* pv. *oryzae*). ZH11 was used as a sensitive control, while Tiankang B was used as a resistance control. (q, r) Phenotypes (q) and lesion length/plant height (r) of rice seedlings infiltrated with *Rhizoctonia solani*. Lemont was used as a sensitive control, while Teqing was used as a resistance control. Scale bars in (a), 2 cm; scale bars in (m), 1 cm; scale bars in the rest, 5 cm. Data are shown as mean ± SD, *n* = 10 in (n), and *n* = 3 for the rest, **p < 0.01, ***p < 0.001.

**Figure S4:** Homology analysis of RGA4L. (a) Protein sequence alignment of RGA4L and its homologues/orthologs in rice and maize. ZmRp1‐D, ZmRp1‐D21 and ZmRp1‐dp2 are the orthologous NLR proteins in maize. OsRGA4 (*LOC_Os11g11790*) and OsRGA5 (*LOC_Os11g11810*) are the homologues of RGA4L in rice. The CC (coiled‐coil), NB (nucleotide‐binding), ARC1 (abbreviated from Apaf‐1, R proteins and CED‐4), ARC2 and LRR (leucine‐rich‐repeat) domains are indicated by the lines with different colours on the top of the alignment, respectively. The motifs (EDVID, P‐loop, RNBS‐A, Walker B, RNBS‐D, MHD1, and MHD2) are indicated by red lines. The conserved amino acid residues related to the function of NLR protein are indicated by red triangles. (b) Phylogenetic tree showing the evolutionary relationships of RGA4L among the monocotyledons.

**Figure S5:** Expression pattern and hypersensitivity response analysis of *RGA4L*. (a) Expression of *DREB1A*, *B*, and *C* in the leaves of Koshi‐NIL‐*qCSS12* ^*jap*^ and ‐*qCSS12* ^*ind*^. Actin was used as a reference gene. The value before chilling stress treatment (0 h) was normalised as 1. (b) Schematic showing the plasmids containing the domains of RGA4L^ind^. The positions of the amino acids defining the domain boundaries are indicated at the top. ARC, abbreviated from Apaf‐1, R proteins and CED‐4; CC, coiled‐coil; CN, contains CC and NB‐ARC domains; FL, full‐length; LRR, leucine‐rich‐repeat; NB, nucleotide binding; NL, contains NB‐ARC and LRR domains. (c) The HR induction (+) or not (−) for each construction corresponding to (b) is listed. (d) Detection of the indicated proteins by western blot. (e) Detection GFP signal of GFP‐fused truncated domains of RGA4L expressed in tobacco leaves. Scale bars, 50 μm. (f) Trypan blue staining of the tobacco leaves at 5 days post infiltration (dpi). Empty vector (EV) infiltrated on the left side was used as a negative control, and the right side infiltrated with the plasmids containing the truncated domains of RGA4L^ind^ protein (15 replicates each). Scale bars, 1 cm.

**Figure S6:** Sub‐localisation of RGA4L. Subcellular localisation of RGA4L^jap^ in rice protoplasts under 28°C (left) and 4°C (right). RGA4L^jap^ fused with GFP emited green fluorescence, while red fluorescence represents mCherry‐fused nucleus marker NLS. Empty vector was used as a negative control. Scale bars, 5 μm.

**Figure S7:** Protein structure of RGA4L. (a) Protein structure of RGA4L^jap^. The red arrow indicates the flexible random coil structure formed at the C‐terminal. (b) Protein structure of RGA4L^ind^. The red arrow indicates the alpha‐helix structure formed at the C‐terminal.

**Figure S8:** OsHSP90 and OsLEA5 physically interact with RGA4L. (a–c) Interaction assay of RGA4L^jap^ and RGA4L^ind^ with OsHSP90/OsLEA5 by LCA in tobacco leaves. nLuc‐BAK1/cLuc‐RbohD and nLuc‐OsSUS1/cLuc‐OsBURP3 were used as the negative and positive controls, respectively. (d–f) Interaction assay of RGA4L^jap^ and RGA4L^ind^ with OsHSP90/OsLEA5 by BiFC in rice protoplasts. Empty vector containing the N‐terminal or C‐terminal of mVenus (mVN or mVC) was used as the negative control. (g, h) Interaction of RGA4L^jap^ and RGA4L^ind^ with OsHSP90/OsLEA5 was tested by pull‐down assay.

**Figure S9:** Subcellular localisation and co‐localisation of RGA4L^jap^, OsHSP90 and OsLEA5 using rice protoplasts. (a) Subcellular localisation of OsLEA5. Green fluorescence represents GFP‐fused OsLEA5, while red fluorescence indicates mCherry‐fused nuclear marker NLS. Empty vector was used as a negative control. (b) Co‐localisation of RGA4L and OsHSP90 in the endoplasmic reticulum (ER). Green fluorescence represents GFP‐fused RGA4L^jap^ and OsHSP90, respectively, while red fluorescence indicates RFP‐fused endoplasmic reticulum marker HDEL. Empty vector was used as a negative control. Scale bars in (a, b), 5 μm.

**Figure S10:** Combined haplotype analysis of 5 chilling tolerance‐related genes. (a) Schematic of the allelic variations of 5 genes from 4726 rice accessions. The chilling‐tolerant or ‐sensitive alleles of the 5 genes are shown with yellow or green shading, respectively. Red and blue font SNPs represent the genotype group of Koshihikari and Nona Bokra, respectively. (b) Histogram shows the survival rate of the genotype groups after chilling stress. (c–e) Pie charts show the proportion of the indicated genotype groups in all examined rice varieties (c), *japonica* (d), and *indica* (e) cultivars. GJ, *japonica* rice; XI, xian_*indica* rice.

**Figure S11:** Yield performance of the *indica* background NILs of *RGA4L*. (a–c) Statistical results of the yield‐related traits of the NJ11 (a), HHZ (b), and 556 (c) background NILs of *RGA4L* at the maturation stage. Data are shown as mean ± SD, *n* ≥ 10, *p < 0.05, ***p < 0.001.

**Data S1:** pbi70293‐sup‐0012‐DataS1.docx.

Table S1:

Table S2:

Table S4:

Table S5:

Table S7:

Table S8:

## Data Availability

The data that supports the findings of this study are available in the Supporting Information of this article.
