# Supplementary figures and images for: Natural Variation of a Specific NLR Gene RGA4L Confers Strong Chilling Tolerance in Rice
Source: Plant Biotechnol J. 2025 Aug 6;23(11):5161–77. doi: 10.1111/pbi.70293 (PMC12576433; doi:10.1111/pbi.70293)

**(a)** Genome-wide scanning of the loci for chilling sensitive phenotype of the CSSLs

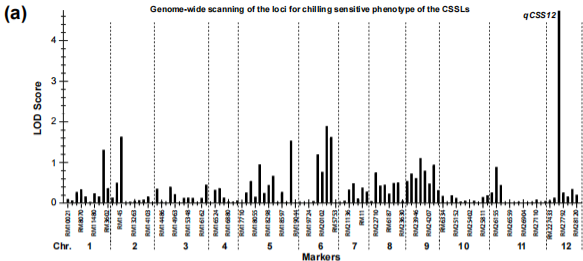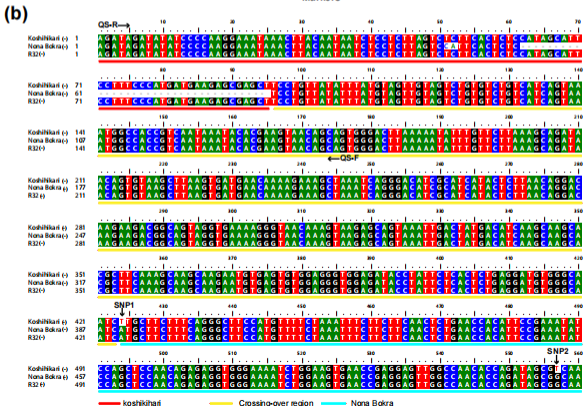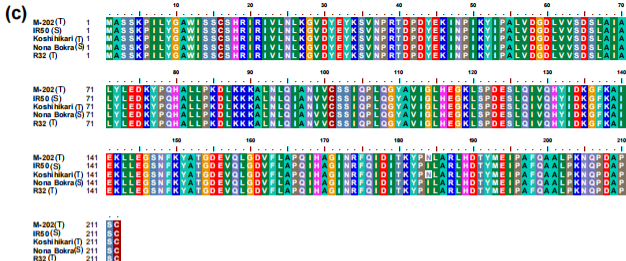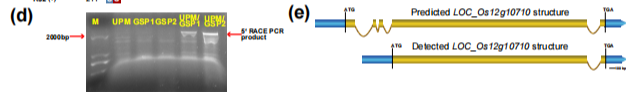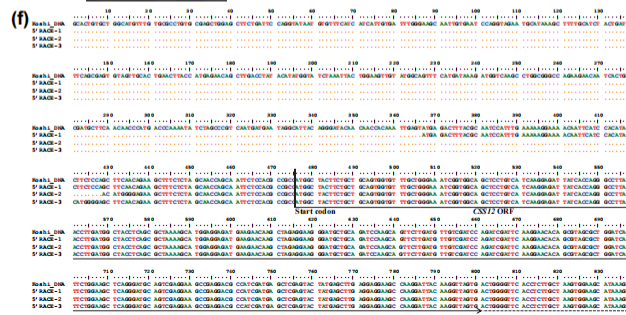

Supplement: Supplementary file 1 — Figure S1: Determination of qCSS12 and the cDNA of its target. (a) Genome‐wide scanning of the QTL loci for chilling sensitive phenotypes of the CSSLs. The x‐axis denotes the location of the molecular markers on chromosomes, and the y‐axis indicates the LOD Score. (b) Sequence alignment of the crossing‐over region in the RGA4L gene. Only the partial sequences flanking the crossing‐over region are shown. The red underline indicates the partial fragment derived from Koshihikari RGA4L jap allele, the yellow underline indicates the crossing‐over region, and the blue underline indicates the partial fragment derived from the Nona Bokra RGA4L ind allele. QS, SNP1, and SNP2 represent the molecular markers for determining the alleles of RGA4L jap /RGA4L ind in R32. (c) Sequence alignment of OsGSTZ2 protein. I99V and N184I are the two causal variants that accounted for chilling stress phenotype variations conferred by qCTS12 (Kim et al. 2011). (d) Agarose gel electrophoresis showing RACE PCR amplification products. GSP, gene specific primer; M, marker; S, chilling sensitive; T, chilling tolerant; UPM, universal primer. (e) Predicted and detected structure of RGA4L gene. (f) Sequence alignment of RACE amplification products. [file PBI-23-5161-s009.pdf]

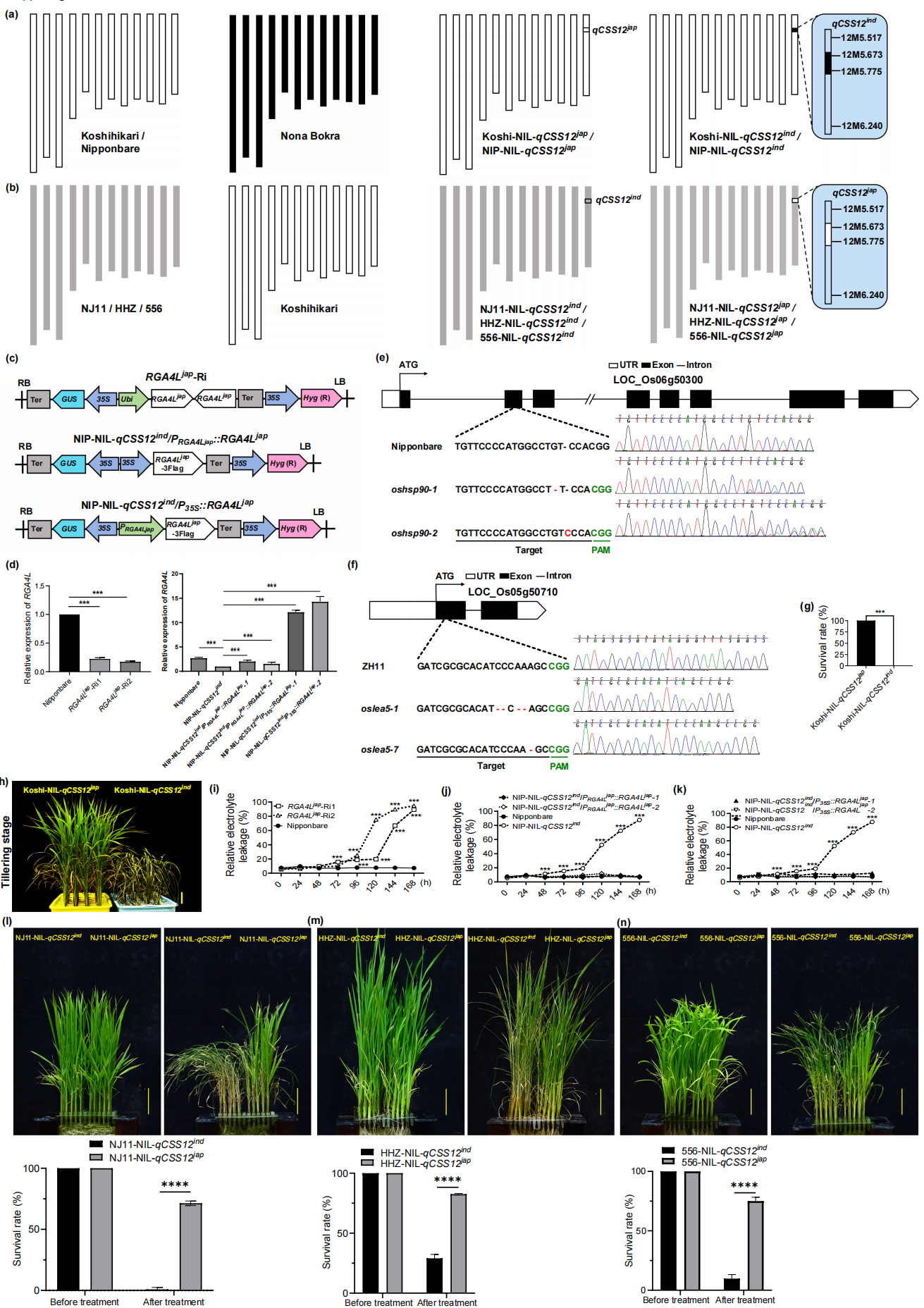

Supplement: Supplementary file 2 — Figure S2: Construction of the NILs and transgenic lines in this study. (a) NILs of qCSS12 constructed in the japonica rice. The black bars show the homozygous fragments derived from Nona Bokra, and the white bars indicate the homozygous regions of the Koshihikari or Nipponbare. Fragment substitution sites are shown in the blue box. (b) NILs of qCSS12 constructed in the indica rice. The white bars show the homozygous fragments derived from Koshihikari, and the grey bars indicate the homozygous regions of the NJ11, HHZ, or 556. Fragment substitution sites are shown in the blue box. (c, d) Generation of the RGA4L transgenic lines. (c) Schematic of the plasmids constructed for generating RNA interference, complementary, and overexpression lines of RGA4L. (d) Expression levels of RGA4L in transgenic lines and wild type. The expression levels in wild‐types Nipponbare (background of RNA interference lines) and NIP‐NIL‐qCSS12 ind (background of complementary lines and overexpression lines) are set as ‘1’. Data are shown as mean ± SD, n = 6, ***p < 0.001. (e) Knockout of OsHSP90 by CRISPR/Cas9 editing technology. (f) Knockout of OsLEA5 by CRISPR/Cas9 editing technology. (g, h) Survival rate (g) and phenotypes (h) of Koshi‐NIL‐qCSS12 jap and ‐qCSS12 ind at the tillering stages. (i–k) Electrolyte leakage of the RGA4L RNAi lines (i), the RGA4L complementary lines (j), and the RGA4L overexpressing lines (k). (l–n) Phenotypes and survival rate of indica background NILs. Scale bars in (h, l–n), 5 cm. Data are shown as mean ± SD, n = 3 in (l, m), ***p < 0.001. [file PBI-23-5161-s006.pdf]

Suppl. Fig. 3

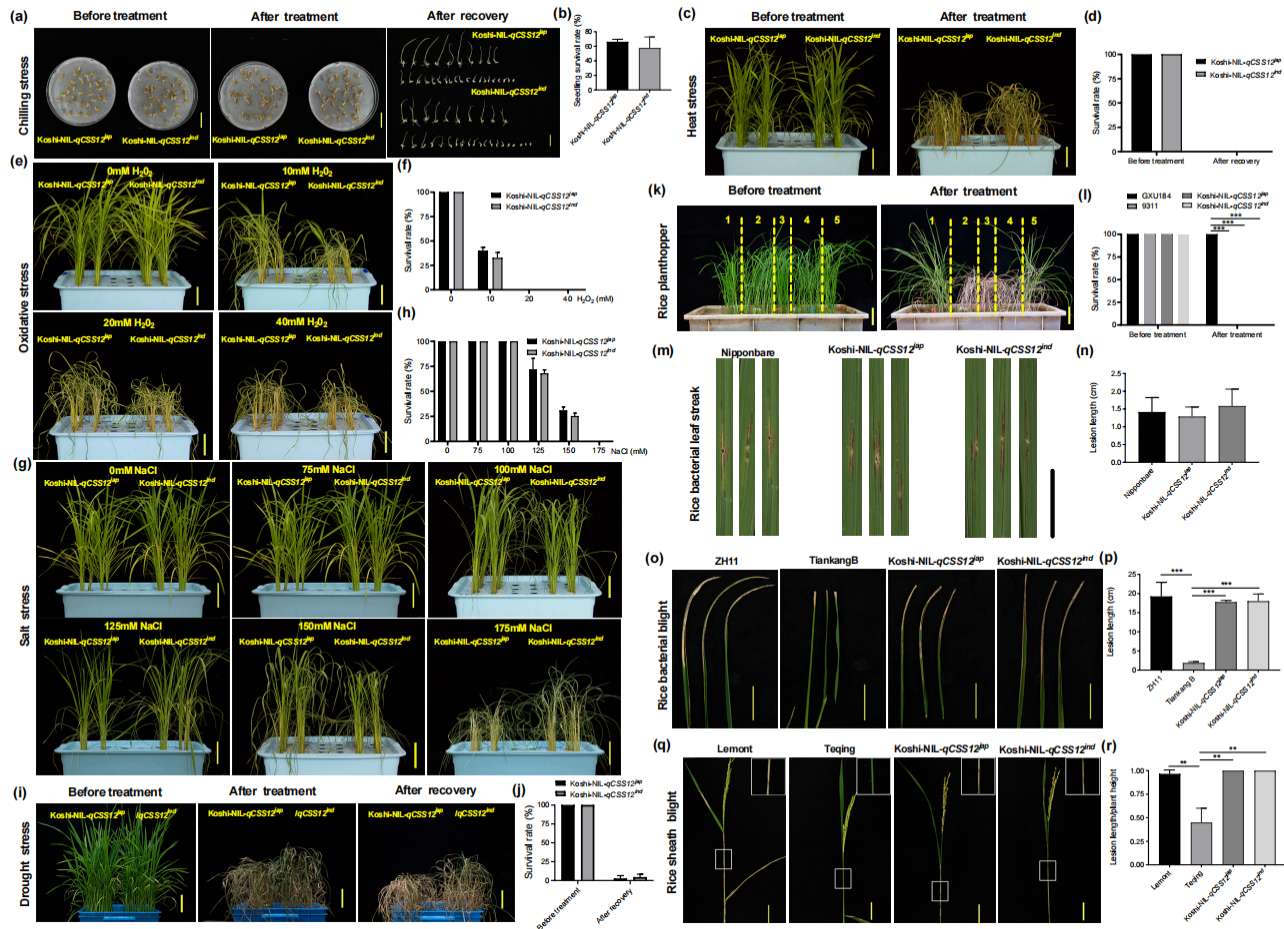

Supplement: Supplementary file 3 — Figure S3: Phenotypes of RGA4L in response to abiotic and biotic stresses. (a, b) Chilling stress phenotypes (a) and seedling survival rate (b) of Koshi‐NIL‐qCSS12 jap and ‐qCSS12 ind at the budding stage. (c–j) Phenotypes and survival rate of Koshi‐NIL‐qCSS12 jap and ‐qCSS12 ind seedlings under heat (c, d), oxidative (e, f), salt (g, h), and drought (i, j) stress. (k, l) Phenotypes (k) and survival rate (l) of rice seedlings infested with planthoppers for 14 days. 1 and 5, GXU184, which were used as a resistance control; 2, Koshi‐NIL‐qCSS12 jap ; 3, 9311, as a sensitive control; 4, Koshi‐NIL‐qCSS12 ind . (m, n) Phenotypes (m) and lesion length (n) of rice leaves infiltrated with Xoc ( X. oryzae pv. oryzicola). Nipponbare was used as a sensitive control. (o, p) Phenotypes (o) and lesion length (p) of rice leaves infiltrated with Xoo ( X. oryzae pv. oryzae). ZH11 was used as a sensitive control, while Tiankang B was used as a resistance control. (q, r) Phenotypes (q) and lesion length/plant height (r) of rice seedlings infiltrated with Rhizoctonia solani. Lemont was used as a sensitive control, while Teqing was used as a resistance control. Scale bars in (a), 2 cm; scale bars in (m), 1 cm; scale bars in the rest, 5 cm. Data are shown as mean ± SD, n = 10 in (n), and n = 3 for the rest, **p < 0.01, ***p < 0.001. [file PBI-23-5161-s018.pdf]

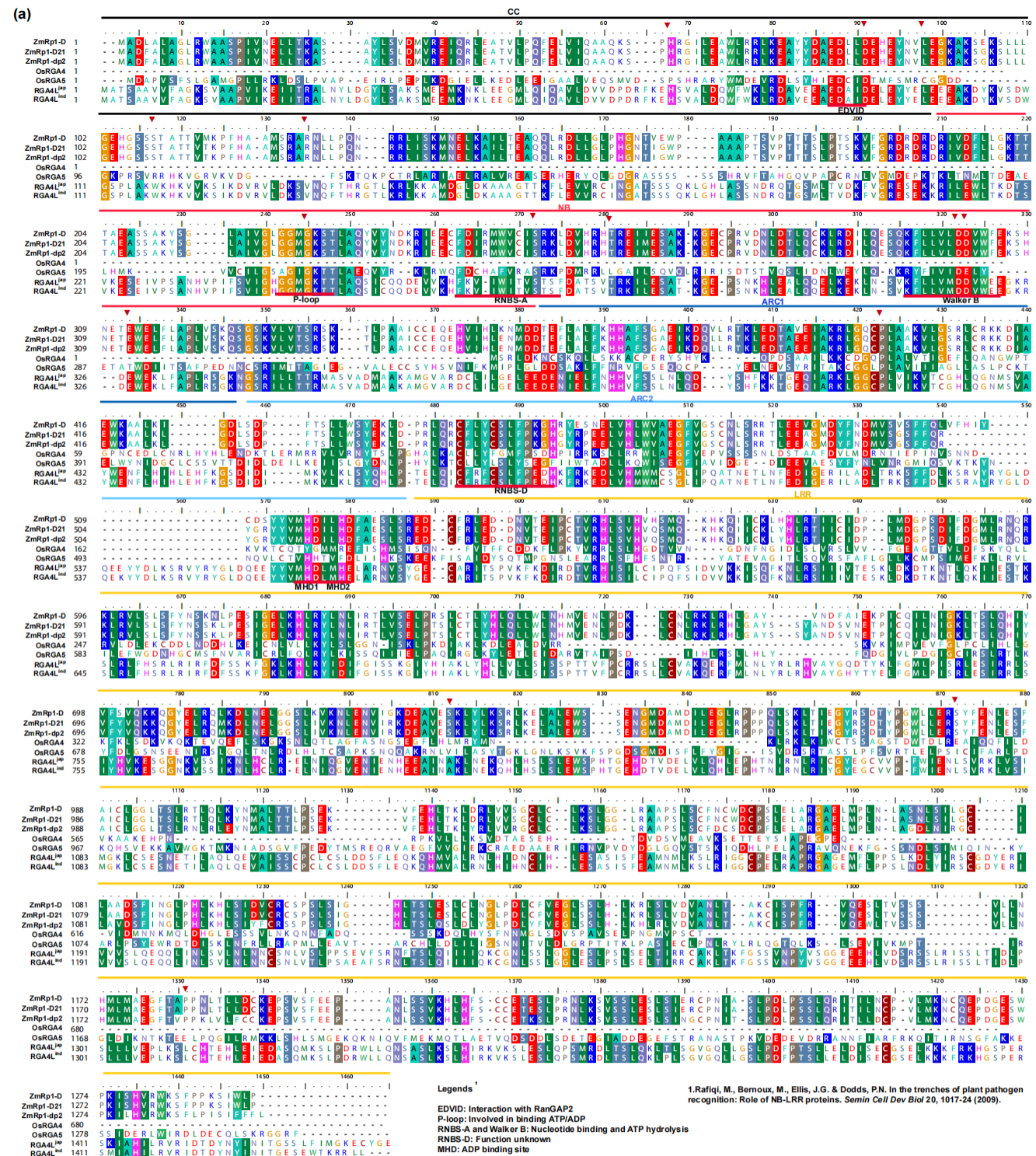**(b)**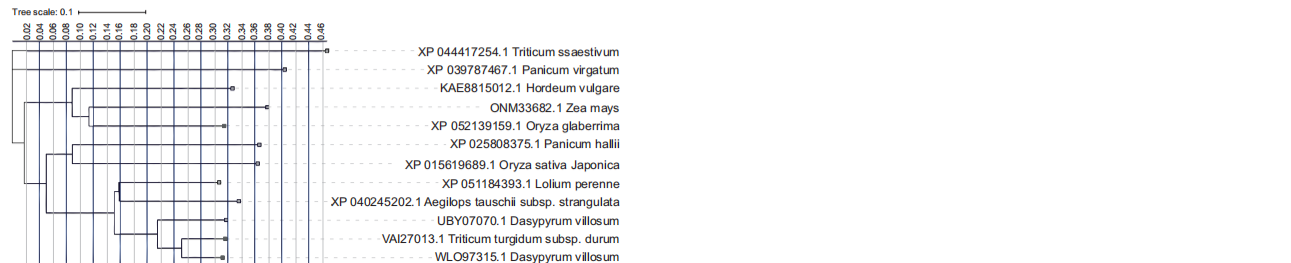

Supplement: Supplementary file 4 — Figure S4: Homology analysis of RGA4L. (a) Protein sequence alignment of RGA4L and its homologues/orthologs in rice and maize. ZmRp1‐D, ZmRp1‐D21 and ZmRp1‐dp2 are the orthologous NLR proteins in maize. OsRGA4 (LOC_Os11g11790) and OsRGA5 (LOC_Os11g11810) are the homologues of RGA4L in rice. The CC (coiled‐coil), NB (nucleotide‐binding), ARC1 (abbreviated from Apaf‐1, R proteins and CED‐4), ARC2 and LRR (leucine‐rich‐repeat) domains are indicated by the lines with different colours on the top of the alignment, respectively. The motifs (EDVID, P‐loop, RNBS‐A, Walker B, RNBS‐D, MHD1, and MHD2) are indicated by red lines. The conserved amino acid residues related to the function of NLR protein are indicated by red triangles. (b) Phylogenetic tree showing the evolutionary relationships of RGA4L among the monocotyledons. [file PBI-23-5161-s016.pdf]

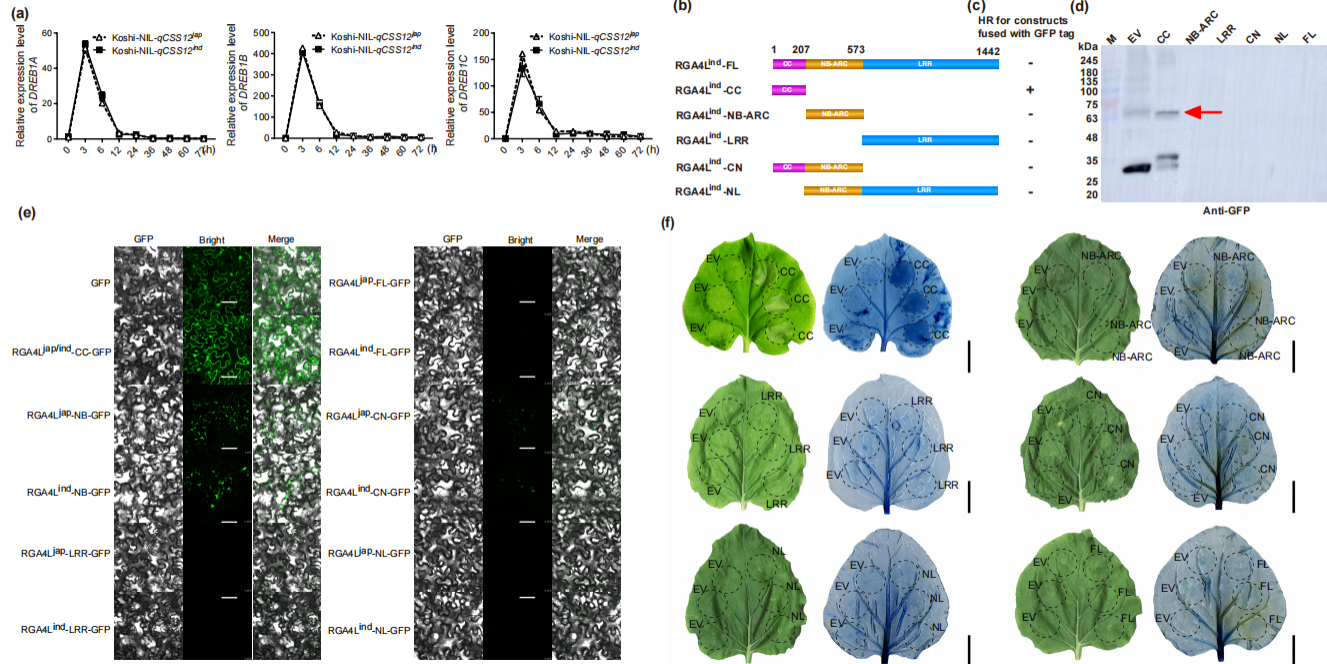

Supplement: Supplementary file 5 — Figure S5: Expression pattern and hypersensitivity response analysis of RGA4L. (a) Expression of DREB1A, B, and C in the leaves of Koshi‐NIL‐qCSS12 jap and ‐qCSS12 ind . Actin was used as a reference gene. The value before chilling stress treatment (0 h) was normalised as 1. (b) Schematic showing the plasmids containing the domains of RGA4Lind. The positions of the amino acids defining the domain boundaries are indicated at the top. ARC, abbreviated from Apaf‐1, R proteins and CED‐4; CC, coiled‐coil; CN, contains CC and NB‐ARC domains; FL, full‐length; LRR, leucine‐rich‐repeat; NB, nucleotide binding; NL, contains NB‐ARC and LRR domains. (c) The HR induction (+) or not (−) for each construction corresponding to (b) is listed. (d) Detection of the indicated proteins by western blot. (e) Detection GFP signal of GFP‐fused truncated domains of RGA4L expressed in tobacco leaves. Scale bars, 50 μm. (f) Trypan blue staining of the tobacco leaves at 5 days post infiltration (dpi). Empty vector (EV) infiltrated on the left side was used as a negative control, and the right side infiltrated with the plasmids containing the truncated domains of RGA4Lind protein (15 replicates each). Scale bars, 1 cm. [file PBI-23-5161-s005.pdf]

Suppl. Fig. 6

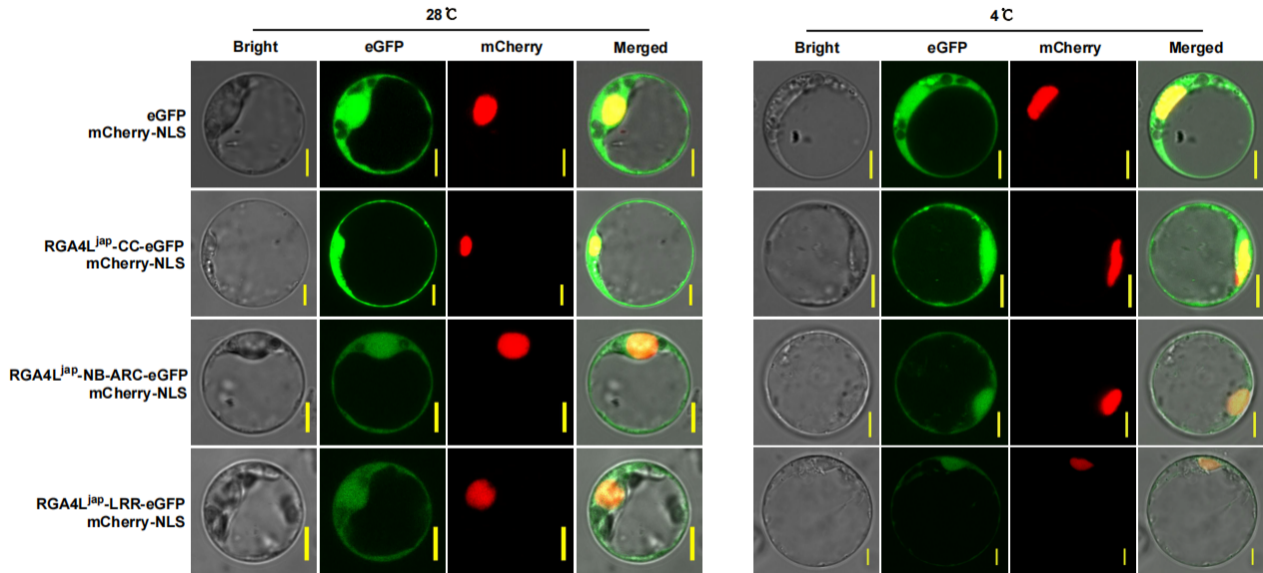

Supplement: Supplementary file 6 — Figure S6: Sub‐localisation of RGA4L. Subcellular localisation of RGA4Ljap in rice protoplasts under 28°C (left) and 4°C (right). RGA4Ljap fused with GFP emited green fluorescence, while red fluorescence represents mCherry‐fused nucleus marker NLS. Empty vector was used as a negative control. Scale bars, 5 μm. [file PBI-23-5161-s015.pdf]

Suppl. Fig. 7

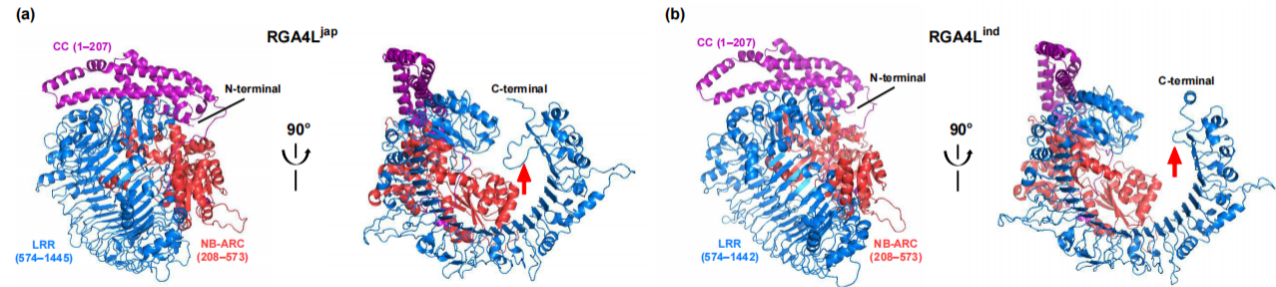

Supplement: Supplementary file 7 — Figure S7: Protein structure of RGA4L. (a) Protein structure of RGA4Ljap. The red arrow indicates the flexible random coil structure formed at the C‐terminal. (b) Protein structure of RGA4Lind. The red arrow indicates the alpha‐helix structure formed at the C‐terminal. [file PBI-23-5161-s011.pdf]

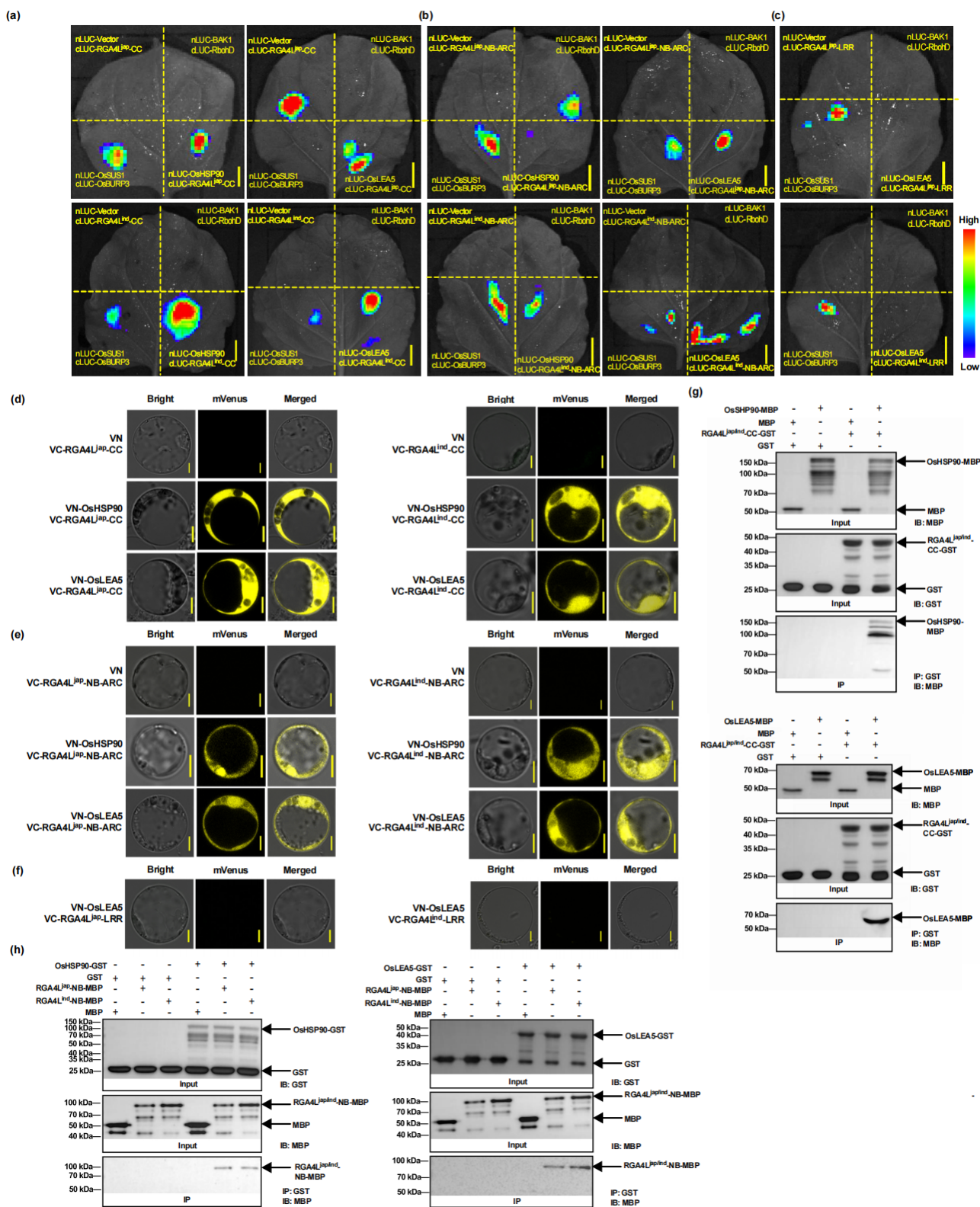

Supplement: Supplementary file 8 — Figure S8: OsHSP90 and OsLEA5 physically interact with RGA4L. (a–c) Interaction assay of RGA4Ljap and RGA4Lind with OsHSP90/OsLEA5 by LCA in tobacco leaves. nLuc‐BAK1/cLuc‐RbohD and nLuc‐OsSUS1/cLuc‐OsBURP3 were used as the negative and positive controls, respectively. (d–f) Interaction assay of RGA4Ljap and RGA4Lind with OsHSP90/OsLEA5 by BiFC in rice protoplasts. Empty vector containing the N‐terminal or C‐terminal of mVenus (mVN or mVC) was used as the negative control. (g, h) Interaction of RGA4Ljap and RGA4Lind with OsHSP90/OsLEA5 was tested by pull‐down assay. [file PBI-23-5161-s002.pdf]

Suppl. Fig. 9

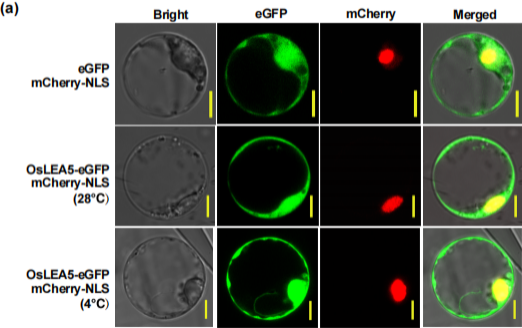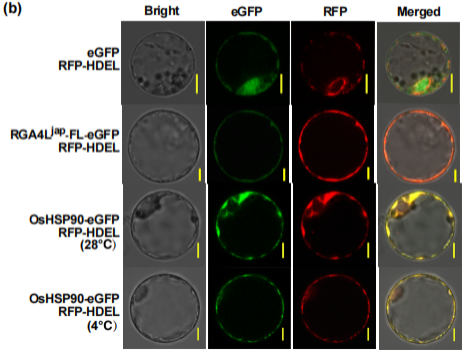

Supplement: Supplementary file 9 — Figure S9: Subcellular localisation and co‐localisation of RGA4Ljap, OsHSP90 and OsLEA5 using rice protoplasts. (a) Subcellular localisation of OsLEA5. Green fluorescence represents GFP‐fused OsLEA5, while red fluorescence indicates mCherry‐fused nuclear marker NLS. Empty vector was used as a negative control. (b) Co‐localisation of RGA4L and OsHSP90 in the endoplasmic reticulum (ER). Green fluorescence represents GFP‐fused RGA4Ljap and OsHSP90, respectively, while red fluorescence indicates RFP‐fused endoplasmic reticulum marker HDEL. Empty vector was used as a negative control. Scale bars in (a, b), 5 μm. [file PBI-23-5161-s010.pdf]

(a)

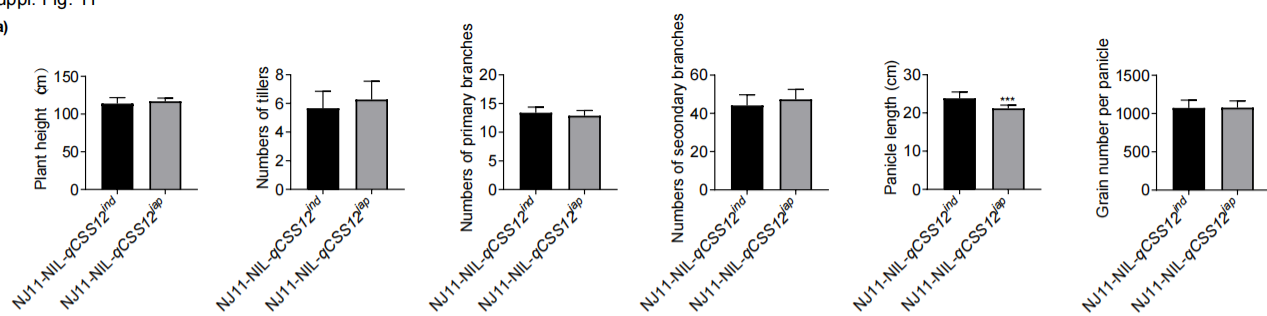

(b)

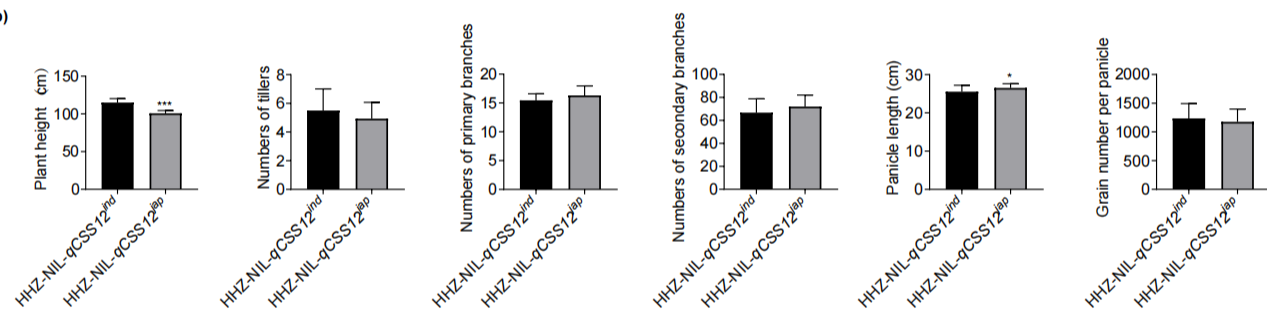

(c)

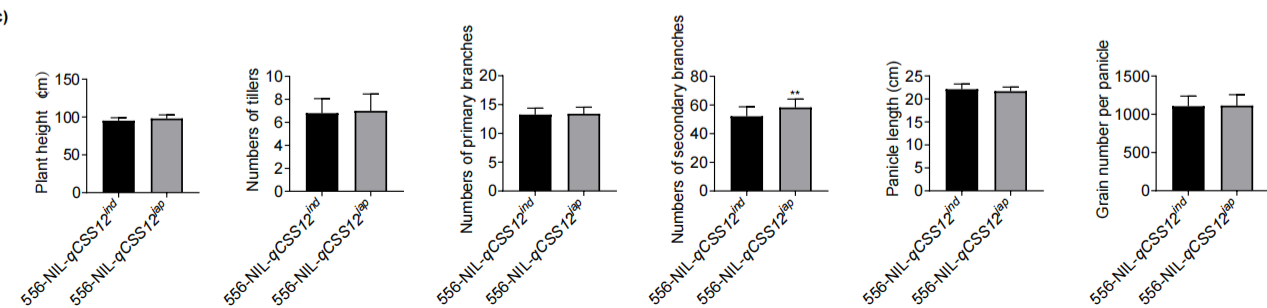

Supplement: Supplementary file 11 — Figure S11: Yield performance of the indica background NILs of RGA4L. (a–c) Statistical results of the yield‐related traits of the NJ11 (a), HHZ (b), and 556 (c) background NILs of RGA4L at the maturation stage. Data are shown as mean ± SD, n ≥ 10, *p < 0.05, ***p < 0.001. [file PBI-23-5161-s017.pdf]
