# Supplementary material for: Natural Variation of a Specific NLR Gene RGA4L Confers Strong Chilling Tolerance in Rice
Source: Plant Biotechnol J. 2025 Aug 6;23(11):5161–77. doi: 10.1111/pbi.70293 (PMC12576433; doi:10.1111/pbi.70293)

Suppl. Fig. 10

(a)

Genotype	Gene Group	CTB4a				COLD1	bZIP73	HAN1	RGA4L	All frequencies	GJ	XI	Aus	Intermediate
		402032301	402032882	402034032	402036871	430315214	918122850	1116984309	1205746769					
Group 1	*CTB4a*^{KMXBG}-*COLD1*^{jap}-*bZIP73*^{jap}-*HAN1*^{02408}-*RGA4L*^{jap}	T	G	A	T	T	C	A	+34 bp	149	149	0	0	0
Group 2	*CTB4a*^{Towada}-*COLD1*^{jap}-*bZIP73*^{jap}-*HAN1*^{02408}-*RGA4L*^{jap}	C	C	G	C	T	C	A	+34 bp	379	378	0	0	1
Group 3	*CTB4a*^{KMXBG}-*COLD1*^{ind}-*bZIP73*^{jap}-*HAN1*^{Teqing}-*RGA4L*^{jap}	T	G	A	T	A	C	G	+34 bp	89	88	0	0	1
Group 4	*CTB4a*^{Towada}-*COLD1*^{ind}-*bZIP73*^{ind}-*HAN1*^{Teqing}-*RGA4L*^{jap}	C	C	G	C	A	T	G	+34 bp	1281	1	1151	56	73
Group 5	*CTB4a*^{Towada}-*COLD1*^{ind}-*bZIP73*^{ind}-*HAN1*^{Teqing}-*RGA4L*^{ind}	C	C	G	C	A	T	G	-	1351	0	1338	7	6
Group 6	*CTB4a*^{KMXBG}-*COLD1*^{jap}-*bZIP73*^{jap}-*HAN1*^{Teqing}-*RGA4L*^{jap}	T	G	A	T	T	C	G	+34 bp	57	56	0	0	1
Group 7	*CTB4a*^{Towada}-*COLD1*^{jap}-*bZIP73*^{jap}-*HAN1*^{Teqing}-*RGA4L*^{jap}	C	C	G	C	T	C	G	+34 bp	195	175	12	0	8
Group 8	*CTB4a*^{Towada}-*COLD1*^{ind}-*bZIP73*^{jap}-*HAN1*^{Teqing}-*RGA4L*^{jap}	C	C	G	C	A	C	G	+34 bp	421	322	62	4	33
Group 9	*CTB4a*^{Towada}-*COLD1*^{ind}-*bZIP73*^{jap}-*HAN1*^{Teqing}-*RGA4L*^{jap}	T	C	G	C	A	C	G	+34 bp	63	60	3	0	0
Group 10	*CTB4a*^{Towada}-*COLD1*^{jap}-*bZIP73*^{jap}-*HAN1*^{02408}-*RGA4L*^{jap}	T	C	G	C	T	C	A	+34 bp	70	70	0	0	0

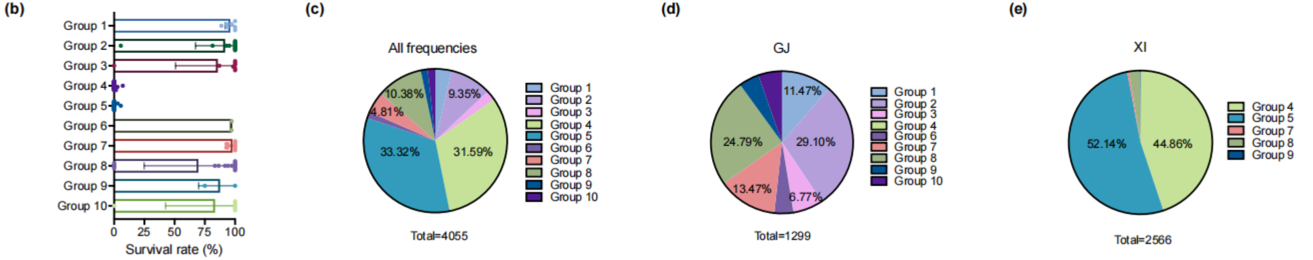

Supplement: Supplementary file 10 — Figure S10: Combined haplotype analysis of 5 chilling tolerance‐related genes. (a) Schematic of the allelic variations of 5 genes from 4726 rice accessions. The chilling‐tolerant or ‐sensitive alleles of the 5 genes are shown with yellow or green shading, respectively. Red and blue font SNPs represent the genotype group of Koshihikari and Nona Bokra, respectively. (b) Histogram shows the survival rate of the genotype groups after chilling stress. (c–e) Pie charts show the proportion of the indicated genotype groups in all examined rice varieties (c), japonica (d), and indica (e) cultivars. GJ, japonica rice; XI, xian_indica rice. [file PBI-23-5161-s001.pdf]
