# Supplementary material for: Natural Variation of a Specific NLR Gene RGA4L Confers Strong Chilling Tolerance in Rice
Source: Plant Biotechnol J. 2025 Aug 6;23(11):5161–77. doi: 10.1111/pbi.70293 (PMC12576433; doi:10.1111/pbi.70293)
Supplement: Supplementary file 12 — Data S1: pbi70293‐sup‐0012‐DataS1.docx. [file PBI-23-5161-s014.docx]

**Table S3 Summary of the HR phenotype conferred by transient expression in the tobacco leaves with infiltration of specific domains or domain combinations from RGA4L *in cis* and *trans*.**

| **Domain combinations** | **HR *in cis*** | **HR *in tran*** |
| --- | --- | --- |
| CC | + | N/A |
| NB-ARC | − | N/A |
| LRR | − | N/A |
| CC+NB-ARC | − | + |
| NB-ARC+LRR | − | N/A |
| CC+NB-ARC-LRR | − | + |
| CC+NB-ARC+LRR | − | + |

Note: *In cis*, the two or three listed domains fused in the same molecule in the order; *in trans*, the two or three listed domains are expressed as separate molecules. All constructs are tagged with 3Myc at the N-terminus. +: HR; －: without HR; N/A: not applicable.

**Table S6 Survival rate of *indica* and *japonica* rice varieties with the different *RGA4L* alleles after chilling treatment.**

| **Genotypes** | **Total accession No.** | **Rice subspecies** | **No. (%)** | **Survival rate** | |
| --- | --- | --- | --- | --- | --- |
|  |  |  |  | **＜50%** | **＞50%** |
| *RGA4L^jap^* | 77 | *Temperate japonica* | 28 (96.55%^a^) | 3.75%^b^ | 96.25% |
|  |  | *Tropical japonica* | 32 (88.89%) | 6.25% | 93.75% |
|  |  | *Indica* | 17 (23.94%) | 70.59% | 29.41% |
| *RGA4L^in^* | 59 | *Temperate japonica* | 1 (3.45%) | 100% | 0% |
|  |  | *Tropical japonica* | 4 (11.11%) | 75% | 25% |
|  |  | *Indica* | 54 (76.06%) | 100% | 0% |

Note: ^a^ represents the percentage of rice varieties in a certain genotype group; ^b^ the percentage of rice varieties that exhibit a corresponding grade of survival rate in a certain genotype group.

**Table S9 The estimated parameters of nucleotide diversity and Tajima’s *D* test of *RGA4L* and its flanking regions.**

|  | **Parameter** | **Ruf** | **GJ_tmp** | **GJ_trp** | **GJ (average)** | **XI_1A** | **XI_1B** | **XI_2** | **XI_3** | **XI (average)** |
| --- | --- | --- | --- | --- | --- | --- | --- | --- | --- | --- |
| Upstream 250 kb | *π* | 0.0411 | 0.0018 | 0.0001 | 0.0010 | 0.0353 | 0.0128 | 0.0239 | 0.0063 | 0.0196 |
|  | θ | 0.0380 | 0.0037 | 0.0005 | 0.0021 | 0.0255 | 0.0207 | 0.0234 | 0.0067 | 0.0191 |
|  | Tajima's *D* | 0.4918 | -1.0581 | -1.1092 | -1.0837 | 1.6042 | -1.0969 | 0.2921 | 0.3382 | 0.2844 |
| *RGA4L* | *π* | 0.1410 | 0.0002 | 0.0010 | 0.0006 | 0.1228 | 0.0510 | 0.1955 | 0.0891 | 0.1146 |
|  | θ | 0.1501 | 0.0013 | 0.0071 | 0.0042 | 0.1213 | 0.1165 | 0.1240 | 0.1153 | 0.1193 |
|  | Tajima's *D* | -0.0733 | -1.1704 | -1.9388 | -1.5546 | 0.1694 | -1.8164 | 2.2258 | -0.6472 | -0.0171 |
| Downstream 250 kb | *π* | 0.1713 | 0.0139 | 0.0094 | 0.0117 | 0.0787 | 0.0377 | 0.1228 | 0.0600 | 0.0748 |
|  | θ | 0.1544 | 0.0062 | 0.0361 | 0.0212 | 0.0745 | 0.0777 | 0.0852 | 0.0721 | 0.0774 |
|  | Tajima's *D* | 0.6300 | 3.4235 | -2.0990 | 0.6623 | 0.7413 | -1.4370 | 2.2561 | -0.0774 | 0.3708 |

**Table S10 Nucleotide polymorphism and neutrality tests of *RGA4L*.**

| **Species/subspecies** | **S^a^** | **h^b^** | **π^c^** | **θ^d^** | **D^e^** | **MLHKA^f^** | |  |
| --- | --- | --- | --- | --- | --- | --- | --- | --- |
|  |  |  |  |  |  | **k^g^** | ***P*-value** |  |
| *O.sativa* | 21 | 6 | 0.0013 | 0.0018 | -1.4691 | 0.711 | 0.1613 |  |
| *indica* | 15 | 5 | 0.0012 | 0.0020 | -0.1327 | 0.832 | 0.2003 |  |
| *japonica* | 6 | 3 | 0.0001 | 0.0002 | -2.0352 | **0.056** | **0.0028*** |  |
| *O.rufipogon* | 6 | 5 | 0.0198 | 0.0646 | 1.0674 | 1.021 | 0.2521 |  |

^a^ Number of samples.

^b^ Number of haplotypes.

^c^ Average number of pairwise nucleotide differences per site.

^d^ Watterson’s estimator of θ per base pair.

^e^ Tajima’s D(Tajima, 1989).

^f^ The maximum likelihood Hudson–Kreitman–Aguadé (MLHKA) test(Wright and Charlesworth, 2004) was performed with *O. barthii* as the outgroup and six neutrally evolving rice genes as the reference(Zhu et al., 2007). * *P*< 0.05.

^g^ Selection parameter (k>1 indicates an excess of polymorphism compared with divergence; k<1 indicates the opposite situation).
